# Driving the Model to Its Limit: Profile Likelihood Based Model Reduction

**DOI:** 10.1371/journal.pone.0162366

**Published:** 2016-09-02

**Authors:** Tim Maiwald, Helge Hass, Bernhard Steiert, Joep Vanlier, Raphael Engesser, Andreas Raue, Friederike Kipkeew, Hans H. Bock, Daniel Kaschek, Clemens Kreutz, Jens Timmer

**Affiliations:** 1 Institute of Physics, University of Freiburg, Freiburg im Breisgau, Germany; 2 Merrimack Pharmaceuticals, Boston, MA, United States of America; 3 Department of Gastroenterology, Hepatology and Infectiology, University Hospital Duesseldorf, Duesseldorf, Germany; 4 Center for Biosystems Analysis (ZBSA), University of Freiburg, Freiburg im Breisgau, Germany; 5 BIOSS Centre for Biological Signalling Studies, University of Freiburg, Freiburg im Breisgau, Germany; Texas A&M University College Station, UNITED STATES

## Abstract

In systems biology, one of the major tasks is to tailor model complexity to information content of the data. A useful model should describe the data and produce well-determined parameter estimates and predictions. Too small of a model will not be able to describe the data whereas a model which is too large tends to overfit measurement errors and does not provide precise predictions. Typically, the model is modified and tuned to fit the data, which often results in an oversized model. To restore the balance between model complexity and available measurements, either new data has to be gathered or the model has to be reduced. In this manuscript, we present a data-based method for reducing non-linear models. The profile likelihood is utilised to assess parameter identifiability and designate likely candidates for reduction. Parameter dependencies are analysed along profiles, providing context-dependent suggestions for the type of reduction. We discriminate four distinct scenarios, each associated with a specific model reduction strategy. Iterating the presented procedure eventually results in an identifiable model, which is capable of generating precise and testable predictions. Source code for all toy examples is provided within the freely available, open-source modelling environment Data2Dynamics based on MATLAB available at http://www.data2dynamics.org/, as well as the R packages dMod/cOde available at https://github.com/dkaschek/. Moreover, the concept is generally applicable and can readily be used with any software capable of calculating the profile likelihood.

## Introduction

Mathematical models represent abstractions of reality that aid to understand a phenomenon of interest. Any model is a simplification, which manifests itself as some features being over-represented, while others are absent. It is important to balance model complexity with experimental observation, such that only those aspects of reality on which the observed data has meaningful information are included in the model. Independent of whether modelling is performed by shrinking of an oversized model that was taken as starting point, or by adding complexity to a core model until the data is well-described, the result is usually a model that is larger than it would need to be. In turn, over-parameterisation in light of limited data is expressed through non-identifiability of parameters. On the one hand, this strongly restricts the applicability of statistical and numerical approaches to a small subset of computationally demanding modelling techniques. On the other hand, non-identifiable parameters often lead to imprecise model predictions. Therefore, including model reduction as an additional step in the modelling process ameliorates lack of predictability and restricts model complexity to an appropriate level.

A variety of methods have been published for reducing the complexity of models. In the following, we summarise relevant ideas and concepts available in the literature, as well as the most related methods to the one proposed here.

In the context of dynamic reaction networks, most methods intend elimination of irrelevant time scales. The *Methods of Invariant Manifolds (MIM)* [1, 2] originate from chaos theory and are based on the fact that after a transient phase, the dynamics usually reduce to low-dimensional manifolds in the phase space. However, the mechanistic interpretation of the dynamic states is lost if the state space is redefined in terms of such manifolds. Furthermore, identification of such manifolds requires detailed knowledge about the model parameters, which is usually not available in system biology. Another class of methods is based on the detection of *quasi steady-states (QSS)* [3–6] requiring again prior knowledge about parameter values, which is typically not available or unreliable. These methods reduce complexity by removing states that are approximately constant and/or reactions that are extremely fast. In [7], another method has been introduced which simplifies complex rational rate equations. The paper suggests further reduction steps by removing reaction rates with negligible fluxes as well as redundant terms in the ordinary differential equations (ODEs).

The aforementioned approaches are applied without direct use of experimental data. Therefore, these approaches rely on prior knowledge concerning parameter values, as well as ignoring both parameter uncertainties and the fact that some parameters can compensate the effect of others on observed model predictions. Alternative approaches have been developed which, like the method we suggest, reduce the complexity up to a level which is specified by available experimental data. These methods typically reduce a large model iteratively until a desired level of complexity is obtained. In statistics, this strategy is known as *backward elimination*. A well-established methodology for backward elimination is available in statistical literature for models where analytical mathematical solutions are available, e.g. for linear regression models as summarised e.g. in [8], or for machine learning applications as discussed in [9]. Methods for its application to non-linear models are briefly discussed in the following.

In the non-linear setting, the likelihood can be approximated by the Hessian when a sufficiently large amount of data is incorporated into the estimation process. This requirement however, is usually strongly violated for ODE models and it has been shown that the Hessian typically only provides an unreliable indication of parameter identifiability. Therefore, confidence intervals based on such an approach are typically inappropriate [10]. Nevertheless, methods have been suggested for removing parameters which appear non-identifiable based on evaluation of the Hessian [11]. An additional aspect emerging for non-linear models, namely the existence of local minima, was addressed in [11, 12]. In both publications, candidates for a backward elimination procedure were suggested. These were selected based on the range of parameter estimates over different local minima. However, both approaches require an arbitrary threshold for defining the local minima of interest and are only able to perform single parameter eliminations, thereby missing model reduction steps that involve a combination of several coupled parameters.

In [13], the optimal reduction is obtained by fitting the dynamics of the reduced system for a pre-specified magnitude of reduction, e.g. for given number of remaining fluxes, to the dynamics of the full model. In this way, the local linearisation of the model, which corresponds to a quadratic approximation of the likelihood, is not required. However, the method again does not account for parameter uncertainties as well as for the availability of data.

The *Manifold Boundary Approximation Method (MBAM)* has been presented in [14] and exploits the fact that eigenvalues of the Hessian matrix range over several orders of magnitude. Starting from the direction with the smallest eigenvalue, a geodesic path is calculated until the Hessian becomes singular which corresponds to a boundary in the model prediction space. However, the initial step of this method approximates the likelihood by a quadratic function. The boundaries in the model prediction space obtained via geodesic paths typically involve arbitrary parameter combinations and therefore hampers mechanistic interpretation of a model reduction step and of the resulting reduced model.

In [15], backward elimination was discussed by defining *admissible regions*. Like in the standard backward elimination setting, the model is iteratively reduced by applying a heuristic strategy for testing whether parameters can be set to the bounds of the parameter space, e.g. to zero. This approach is conceptionally the most similar one to our method based on the profile likelihood. One advantage of our method is that it provides a clear strategy for determining which parameters have to be tested for reduction. In addition, inferring model reduction steps involving re-parameterisations of several coupled parameters are feasible.

In summary, the method introduced in the following does not rely on prior knowledge or likelihood approximation by the Hessian. Moreover, it takes into account parameter uncertainties given model and data and features a clear strategy for determining relationships of multiple parameters while preserving a mechanistic interpretation of the reduced model.

## Methods

### The underlying idea

Our method can be motivated by a prominent example from systems biology: consider the conversion

$$S+E\;\overset{k_{+}}{\underset{k_{-}}{⇌}}C\overset{\;k_{2}\;}{\to }P+E$$ (1)

of a substrate *S* into a product *P* by an enzyme *E* via formation of an intermediate complex *C*. The Michaelis-Menten kinetics

$$\dot{P}=k_{2}E\frac{S}{K_{m}+S}$$ (2)

is a frequently used approximation which holds for $\dot{C}\approx 0$. This assumption is satisfied if, for instance, the rates *k*_+_ and *k*_−_ are much larger than *k*_2_. Indeed, in the joint limit *k*_±_ → ∞, under the constraint $\frac{k_{-}}{k_{+}}=const.$, the kinetics of the full model converge to Michaelis-Menten kinetics with $K_{m}=\frac{k_{-}+k_{2}}{k_{+}}\to \frac{k_{-}}{k_{+}}$. In terms of balancing model complexity with the available data, such a simplification would be acceptable as long as no other evidence requires employing a more complex model.

This example summarises concisely the two fundamental principles underlying our model reduction approach. (1) Reduced models emerge in the limit of extreme parameter values—our method constructs and investigates these limits systematically. (2) A reduced model is a valid approximation if it does not contradict the data at hand—our method systematically searches for reductions, which cannot be rejected by statistical testing.

**Fig 1:**
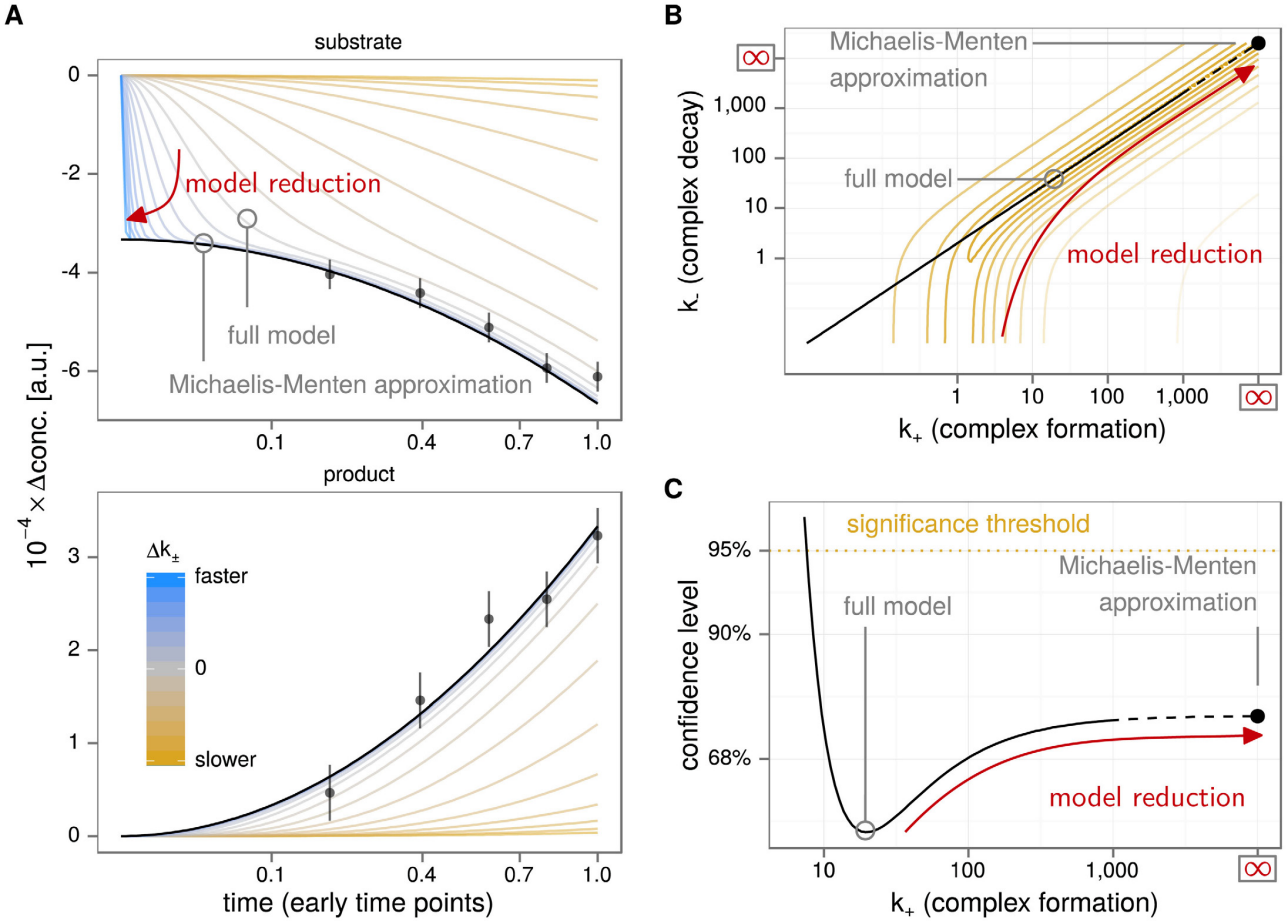
Emergence of the Michaelis-Menten approximation. **A:** Fast complex formation and decay (blue trajectories) result in the Michaelis-Menten approximation, slow formation and decay (orange trajectories) in a significant discrepancy to the data. **B:** The path in parameter space leading to the Michaelis-Menten approximation runs parallel to the contour lines of the log-likelihood function. **C:** The log-likelihood defines a significance threshold, which is not exceeded in the limit of fast formation/decay rates. Slower rates quickly lead to significant deviations from the data.

Starting with the example above, the Michaelis-Menten approximation is obtained in the limit of rapid complex formation and decay compared to the conversion rate. This transition is rendered more precisely in Fig 1. The model response for different values of constant ratio $K_{m}=\frac{k_{+}}{k_{-}}$ is depicted in state space (A), parameter space (B) and the log-likelihood landscape (C). The different scenarios are parameterised by the complex formation rate *k*_+_. For larger values, the dynamics of substrate and product concentrations approach a limiting behaviour indicated by a black line, Fig 1A, which is the prediction derived from Michaelis-Menten kinetics. Based on the hypothetical data points, models with slow complex formation/decay (orange curves in Fig 1A) deviate significantly from the data whereas those with fast complex formation/decay (blue curves) do not. This can be quantified by means of the least squares function or the log-likelihood, shown in Fig 1B and 1C. Every point in *k*_+_-*k*_−_ space is associated with a unique model prediction and a corresponding log-likelihood value to measure the distance from the data points. The model reduction path in this space runs along a valley ending on a log-likelihood level that is not significantly increased in comparison to the optimal parameterisation of Eq (1) (full model). Since the end-point, the Michaelis-Menten approximation, was known, we could easily find this path. Conversely, the particular characteristics of our model reduction method is to construct these paths. This is achieved by computing the profile likelihood [16], which drives the model parameters systematically towards small and large values, respectively, while controlling and minimising the discrepancy between data and model prediction. The required mathematical tools for maximum-likelihood estimation in dynamic mathematical models and the profile likelihood method are introduced in the next sections.

### Mathematical modelling

The rate-equation approach is a well-established methodology employing the mass-action law of chemical reactions for translating biochemical interactions into a mathematical model. Such a model is *mechanistic*, i.e. each component *x* and parameter of the network model has its counterpart within the biological process. Thus, it allows to infer knowledge about the underlying network and drives biological discoveries, e.g. in [17–19]. Furthermore, in [20], an overview about unravelling dynamical features from biological systems by ODE modelling is presented. The time evolution *x*(*t*) of the concentrations of biochemical compounds is computed by solving the corresponding ODE system

$$\dot{x}\left(t\right)=f\left(x\left(t\right),\theta_{x}\right)\;,$$ (3)

with parameters *θ*_*x*_, e.g. reaction rates or Hill coefficients. Initial concentrations *x*_0_ are either fixed using prior assumptions or estimated from the data. The internal states *x*(*t*) are mapped to observations *y*(*t*) via an observation function *g*, i.e.

$$y\left(t\right)=g\left(x\left(t,\theta_{x},x_{0}\right),\theta_{y}\right)+\epsilon \left(t\right)\;,$$ (4)

where independent additive Gaussian errors *ϵ* ∼ *N*(0, *σ*^2^) are assumed. In addition to the dependency on kinetic rates and initial concentrations, the observation function *g* may introduce observational parameters *θ*_*y*_, which are subsumed in *θ* = {*θ*_*x*_, *x*_0_, *θ*_*y*_}. To quantify the discrepancy between model response and measurements, the scaled negative likelihood-function is calculated via

$$\begin{array}{cc} -2\log(L) & :=L\left(\theta \right)=\sum_{i=1}^{n}{\left(\frac{y_{i}-g\left(x\left(t_{i},\theta_{x},x_{0}\right),\theta_{y}\right)}{\sigma_{i}}\right)}^{2}+const.\;, \end{array}$$ (5)

for *n* measured data-points *y*_*i*_ with standard deviation *σ*_*i*_. If the measurement noise is not normally distributed, transformations resulting in Gaussian errors can be applied in most cases. Often, a log-transformation is sufficient [21]. Eq 5 can be amended by penalisation terms representing prior knowledge. These can be e.g. quadratic terms for normally distributed parameters, with mean and standard deviation taken from literature, or for derived model components such as the ratio between protein concentrations in different cell compartments. To estimate model parameters, we apply maximum likelihood [22] by minimising the scaled negative log-likelihood

$$L\left(\hat{\theta }\right)=\min_{\theta }L\left(\theta \right)\;.$$ (6)

leading to an optimised parameter vector $\hat{\theta }$. To ensure positive values and improve numerical performance, all entries of *θ* are optimised on a logarithmic scale throughout the manuscript. Finding the global optimum can be challenging due to the existence of multiple local minima. Therefore, we performed a deterministic multi-start optimisation strategy with widely dispersed initial guesses [23].

In general, no analytical solution of the ODE system (Eq (3)) exists or is available, hence numerical solvers are utilised to approximate the dynamics. Here, we used *CVODES* from the SUNDIALS suite [24] for ODE integration. The minimisation in Eq (6) is performed numerically using the trust-region-based large-scale non-linear optimisation algorithm *lsqnonlin* [25] as implemented in MATLAB. Gradient-based parameter estimation strategies depend on the sensitivities of the model function, i.e. inner derivatives of the likelihood. In order to ensure numerical accuracy, we computed and supplied forward sensitivities [26] by extending the ODE system with the appropriate sensitivity equations. All model analyses, optimisation and visualisation of model responses for the manuscript were performed using the freely available Data2Dynamics modelling environment [27] for MATLAB (http://www.data2dynamics.org/). Additionally, implementations of the toy models and analyses using the freely available dMod/cOde packages for R (https://github.com/dkaschek/) can be found in the Supplementary Section 1.

### Parameter profile likelihood

When fitting models to data, the precision of parameter estimates is assessed by computing confidence intervals. In non-linear models, confidence intervals that are not confined to the lower and/or higher limit of a parameter may occur. In this case, a parameter is termed non-identifiable. In contrast, a parameter with a finite confidence interval is called identifiable.

The profile likelihood is an established concept to assess parameter identifiability in non-linear regression. It generalises Fisher Information based confidence intervals to the non-linear setting, resulting in appropriate confidence regions [10]. In short, a parameter of interest *θ*_*i*_ is profiled by scanning along its axis and re-optimising all other parameters *θ*_*j* ≠ *i*_ for each value of *θ*_*i*_. Thus, the profile likelihood is defined as

$$\text{PL}\left(\theta_{i}\right)=\min_{\theta_{j\neq i}}L\left(\theta \right)\;.$$ (7)

PL(*θ*_*i*_) traces an optimal path through the parameter space for each *θ*_*i*_, thereby providing global information in contrast to the curvature of Eq (5) evaluated at $\hat{\theta }$, as used for Fisher Information based confidence intervals. If $\chi_{\alpha ,1}^{2}$ denotes the *α* quantile of the *χ*^2^ distribution with one degree of freedom, the region for which the inequality

$$D:=\text{PL}\left(\theta_{i}\right)-L\left(\hat{\theta }\right)\leq \chi_{\alpha ,1}^{2}$$ (8)

is satisfied yields the confidence interval of the parameter *θ*_*i*_ to a given confidence level *α*. This means that under weak assumptions [28] (1 − *α*) specifies the probability that, for repeated experiments, the true value of *θ*_*i*_ lies within the boundaries of the confidence interval. $L\left(\hat{\theta }\right)+\chi_{\alpha ,1}^{2}$ defines the threshold that PL(*θ*_*i*_) may not exceed for an acceptable parameter. Thereby, the profile likelihood provides the range of parameter values supported by the available measurement data. The re-optimisation of other parameters during the profile likelihood calculation is crucial to probe the non-linear relationships between parameters, which are key for discovering suitable model reductions later in the manuscript.

Concerning parameter profiles, three scenarios can be distinguished: A parameter can be 1) identifiable, 2) structurally non-identifiable, or 3) practically non-identifiable [10]. Each case is associated with a recognisable shape of the profile likelihood.

An *identifiable* parameter has a confined confidence interval, i.e. the corresponding profile likelihood PL(*θ*_*i*_) exceeds the threshold given by $L\left(\hat{\theta }\right)+\chi_{\alpha ,1}^{2}$ for values above and below its maximum likelihood parameter estimate ${\hat{\theta }}_{i}$. For an identifiable parameter the shape of the profile likelihood is often close to quadratic. This setting is referred to as the *asymptotic* setting, because the profile likelihood based confidence intervals are similar to those calculated from Fisher Information. Thus, asymptotic confidence intervals calculated using only the local curvature of the likelihood at the optimum $\hat{\theta }$ are valid in this scenario.

*Structurally non-identifiable* parameters are characterised by the profile likelihood of *θ*_*i*_ remaining constant for arbitrary values of *θ*_*i*_. Structural non-identifiabilities are a consequence of over-parameterisation in terms of too many parameters for describing the available data. Hence, the system harbours symmetries that can be detected by exploiting Lie group theory [29]. Once the symmetry transformations are found, they allow reconstruction of the full solution space from a reduced set of parameters. Therefore, one parameter of each detected transformation group is set to a fixed value. Thereby, the reduced set of parameters becomes structurally identifiable.

Finally, a non-identifiable parameter characterised by a profile with a (not necessarily unique) global minimum, but which does not exceed the statistical threshold in at least one direction is called *practically non-identifiable*. In such cases, the parameter does not have a finite confidence interval but could be restricted to one side, e.g. the parameter must not be larger than a certain value but could be arbitrarily small. Hence, asymptotic confidence intervals are inappropriate as they are finite by construction. This feature is inherited from the linearity assumption of asymptotic confidence intervals, making practical non-identifiability a property that only occurs in non-linear models. For practically non-identifiable parameters, repeating the same measurements can in principle lead to identifiability, given that the true solution is part of the parameter space. Experimental design based on parameter profiles can be applied to efficiently attain identifiability [30]. However, if new data acquisition is impossible or infeasible, practical non-identifiabilities can be resolved by applying the model reduction technique presented in the following, eventually resulting in a completely identifiable model.

## Results

The reduction procedure described in this paper is based on the parameter profile likelihood introduced above. Identifiable parameters have a well-defined confidence interval and may not be reduced. Structurally non-identifiable parameters can be set to an user-defined value. This leaves practically non-identifiable parameters to serve as starting point for model reduction. Like in the introductory example (Fig 1), the possibility of practically non-identifiable rate constants being driven to infinity implies that the corresponding reactions may be arbitrarily fast and are subsequently *lumped* in a model reduction step.

More general, each point along a parameter profile PL(*θ*_*i*_) can be interpreted as a likelihood-ratio test with the test-statistic *D* according to Eq (8). If a parameter *θ*_*i*_ is practically non-identifiable, the profile likelihood does not exceed the threshold and in most cases flattens out to a constant value in the unbound direction of the confidence interval. The parameter values along the profile likelihood define a sub-manifold in the parameter space, whose respective dynamics correspond to model behaviour which cannot be rejected by the likelihood-ratio test. Similar to the structurally non-identifiable case, the model exhibits a symmetry which can be exploited to reduce the model. Hence, for practically non-identifiable parameters, the profile likelihood method systematically drives the system towards limit behaviour. It provides direct information on the asymptotic coupling/uncoupling of parameters via the parameter paths and reveals the behaviour of the limit model by inspection of the model predictions along the sub-manifold. It is this limit behaviour which ultimately corresponds to the idealised reduced model. In [10], the authors provide a more detailed description on parameter identifiability.

Four different scenarios leading to model reduction can be distinguished. On the one hand, a parameter’s limit behaviour that cannot be rejected by the likelihood-ratio test can occur for parameter values towards plus (+) or minus (−) infinity. On the other hand, the paths of the remaining model parameters can be coupled (↕) to the parameter of interest or remain at the same value (|). This leads to the four scenarios (+|), (−|), (+↕), and (−↕). To make statements more concrete, we chose a representative *in-silico* example for each of these four scenarios. Details about their respective model equations, parameter values, and an extensive model reduction procedure for each example are provided in the Supplementary Section 1. Subsequently, reduction of a biological model of the Reelin signalling cascade is exerted using the presented model reduction strategy based on the profile likelihood.

### Scenario 1: (+|)

**Fig 2:**
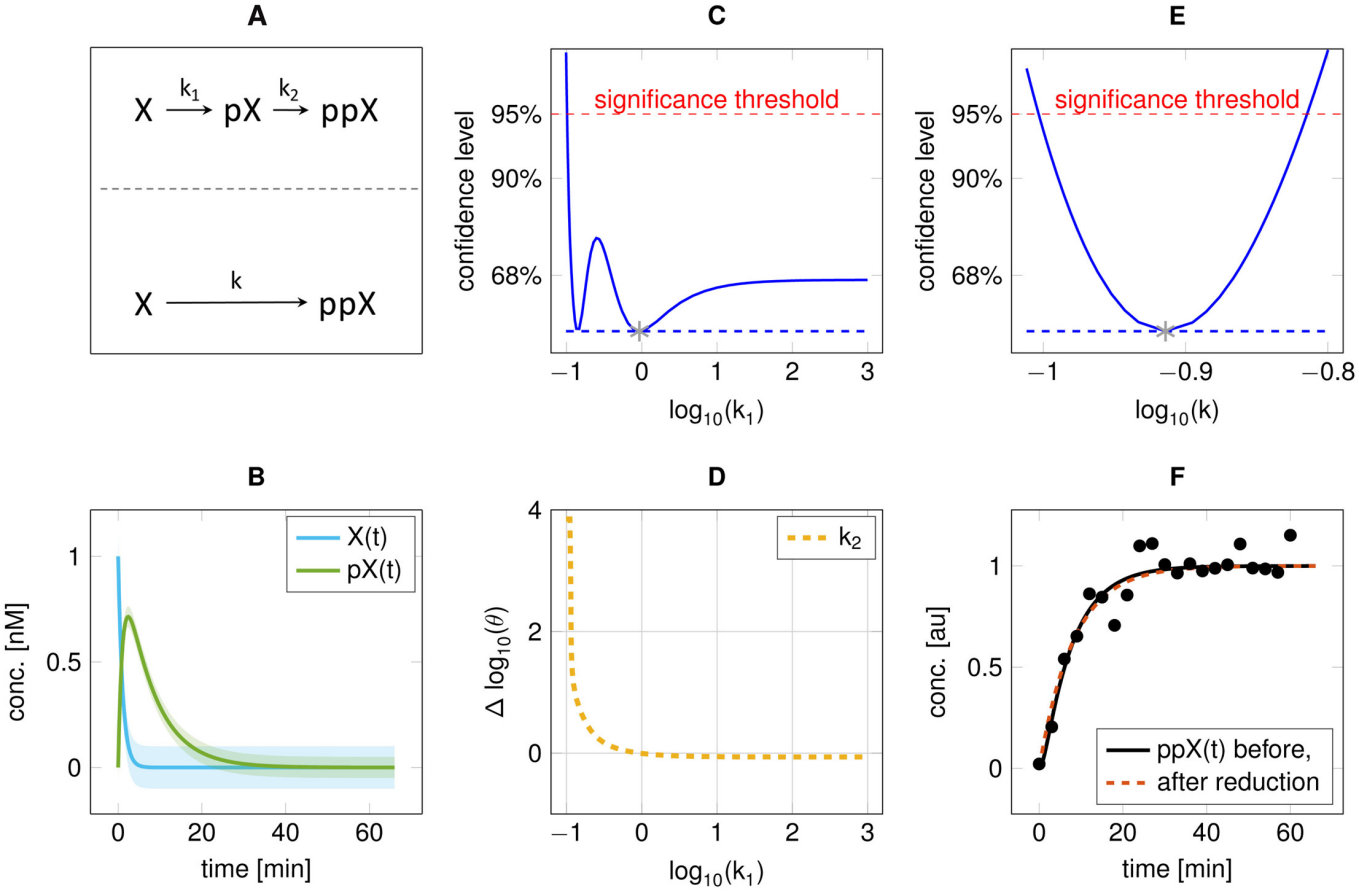
Reduction of the cascade toy model 1 (scenario 1). **A:** Model scheme before (upper) and after (lower) reduction with lumped states *X* and *pX*. **B:** *X*(*t*) and *pX*(*t*) for fitted parameters. **C:** Profile likelihood for parameter *k*_1_. **D:** Relationship of *k*_1_ to *k*_2_ obtained by re-optimisation along the profile likelihood (panel C). **E:** Profile likelihood for the identifiable parameter *k* in the reduced model. **F:** *ppX*(*t*) with simulated data after fitting, with comparison between a one and two step conversion.

The illustrative model used for the basic reductions is a cascade $X\overset{k_{1}}{\to }pX\overset{k_{2}}{\to }ppX$, where each arrow indicates an elementary reaction parameterised by *k*_1_ and *k*_2_, respectively (Fig 2A). The dynamics of *X*(*t*) and *pX*(*t*) are shown in Fig 2B. In this scenario, data is observed for *ppX*(*t*) with *σ* = 0.1 (Fig 2F). By calculating the profile likelihood of the kinetic parameter *k*_1_ towards +∞ two observations can be made. (1) two local optima exist around -1 and 0, which reflect the interchangeability of the reaction rates of two consecutive steps in a conversion chain for unobserved intermediate *pX*. (2) the parameter *k*_1_ is practically non-identifiable as the profile likelihood of *k*_1_ is below the statistical threshold for large values (Fig 2C). Thus, high values of the parameter k1 do not decrease the ability of the model to describe the data. Furthermore, no other parameter is compensating the changes in parameter *k*_1_, which manifests in *k*_2_ flattening out for *k*_1_ → ∞ (Fig 2D). Since a large value for *k*_1_ corresponds to fast conversion of *X* to *pX*, a reduction of the model by *lumping* the states *X* and *pX* is feasible. In the reduced model $X\overset{k}{\to }ppX$, the remaining rate constant is identifiable (Fig 2E), whereas the data is equally well described (Fig 2F).

### Scenario 2: (−|)

**Fig 3:**
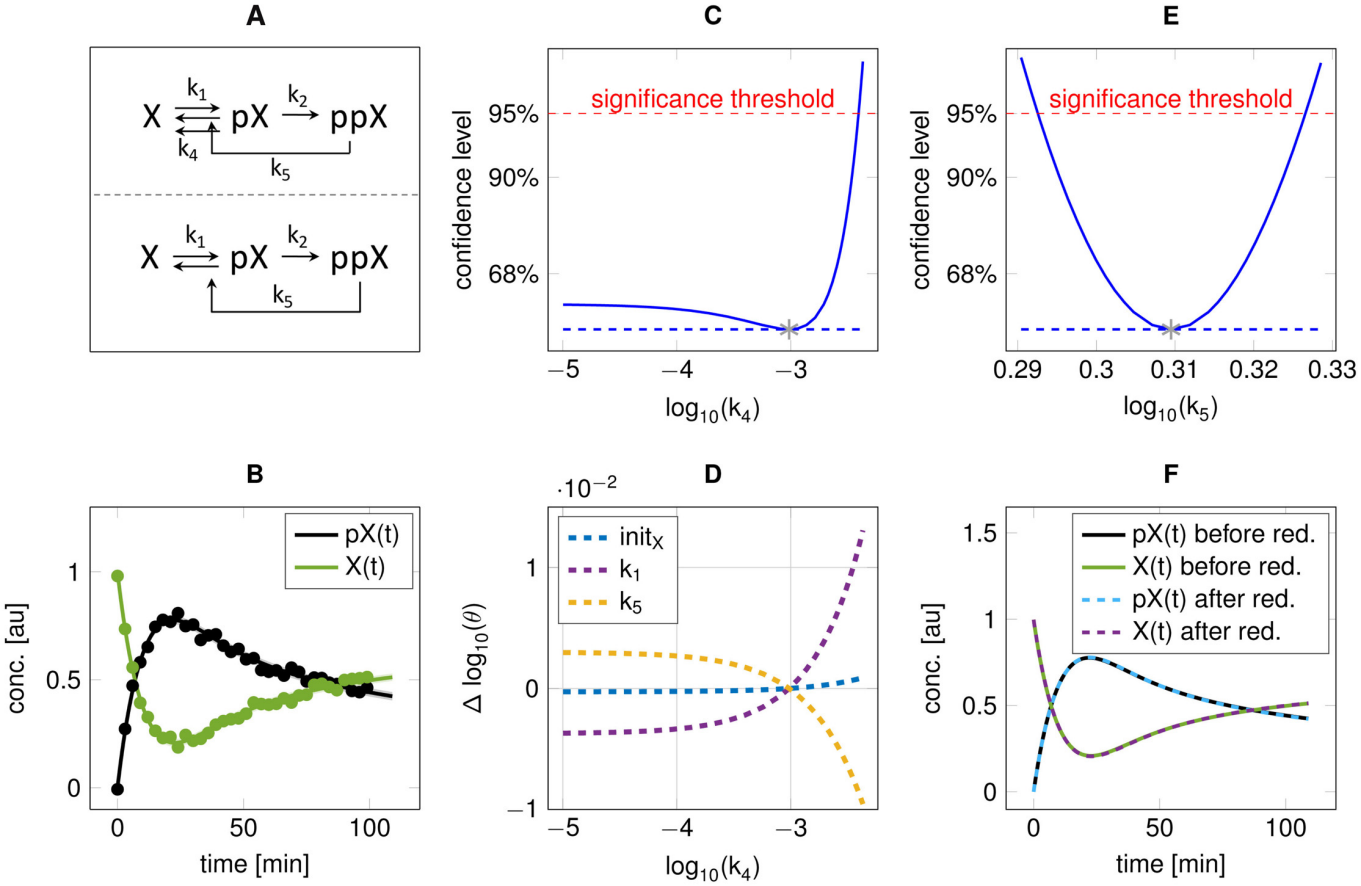
Reduction of the cascade toy model 2 (scenario 2). **A:** Model scheme before (upper) and after (lower) reduction. The difference is the basal deactivation from *pX* to *X* with rate constant *k*_4_. **B:** *X*(*t*) and *pX*(*t*) with data for fitted parameters. **C:** Profile likelihood for parameter *k*_4_. **D:** Relationship of the remaining parameters to the profile shown in C. **E:** The reaction associated with the parameter *k*_4_ is removed by the reduction, while *k*_5_ is identifiable. **F:** *pX*(*t*) and *X*(*t*) with comparison before and after reduction.

Similar to the previous example, we use a model structure featuring a cascade *X* to *pX* and *ppX*, as shown in Fig 3A, with simulated data with *σ* = 0.02 for *X*(*t*) and *pX*(*t*) (Fig 3B). In addition, *pX* is dephosphorylated to *X* with a basal rate, and by a feedback through *ppX*. The profile likelihood for *k*_4_ shows a practical non-identifiability of the basal dephosphorylation, as the lower boundary is not defined (Fig 3C) with constant values of the other parameters as *k*_4_ approaches −∞ (Fig 3D). On the other hand, the parameter *k*_5_, reflecting the dephosphorylation through a feedback by *ppX*, is identifiable as shown by the corresponding profile likelihood (Fig 3E). Here, an appropriate reduction is given by the *removal* of the basal dephosphorylation of *pX* (Fig 3A), with similar dynamics of *pX*(*t*) before and after reduction (Fig 3F).

### Scenario 3: (+↕)

**Fig 4:**
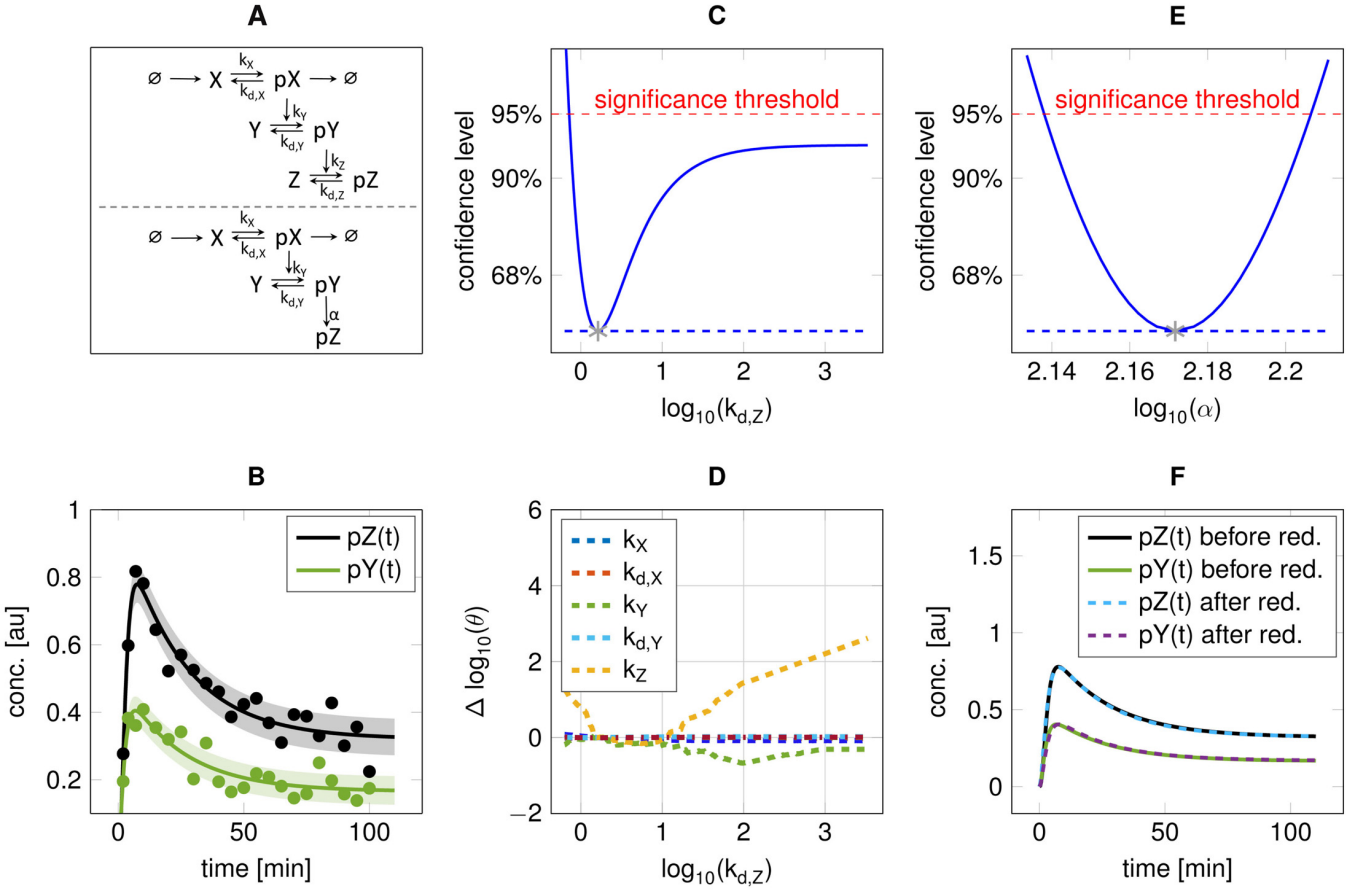
Reduction through functional relation (scenario 3). **A:** Model scheme before (upper) and after (lower) reduction. The difference is the removal of state *Z*, and the algebraic replacement *pZ*(*t*) = *α* ⋅ *pY*(*t*). **B:** *pY*(*t*) and *pZ*(*t*) with simulated data after fitting. **C:** Profile likelihood for parameter *k*_*d*,*Z*_. **D:** Re-optimisation of remaining parameters along the profile likelihood for *k*_*d*,*Z*_. **E:** The profile likelihood for the identifiable parameter *α* in the reduced model. **F:** Comparison of trajectories for *pY*(*t*) and *pZ*(*t*).

In the following, an example of a model is given where the ODE solution of one state can be replaced by an *algebraic equation* (Fig 4A). It features qualitatively similar transient dynamics on a different scale for two successive phosphorylations, *pY*(*t*) and *pZ*(*t*) (Fig 4B). Data is observed with *σ* = 0.05. Since there is no feedback, the activation of state *Z* cannot be faster than the one of state *Y* [31]. If the total amount of *Z* is not limiting, the dynamics *pY*(*t*) and *pZ*(*t*) have similar shape when the phosphorylation and dephosphorylation parameters tend to infinity. In this example, the kinetic parameter for the dephosphorylation of state *Z*, *k*_*d*,*Z*_ is non-identifiable in its upper direction (Fig 4C). The phosphorylation rate *k*_*Z*_ will compensate for changes since $\frac{k_{Z}}{k_{d,Z}}$ determines the final concentration level of state *Z* (Fig 4D). To overcome this practical non-identifiability, the kinetic rate equation of state *Z* is replaced by a simple functional relation given by *Z* = *αY*, which makes *α* identifiable (Fig 4E). The reduced model is able to describe the measurements without a statistically significant decrease in the likelihood compared to the full model (Fig 4F).

For this scenario, a comparison with model reduction based on negligible fluxes is conducted in Supplementary Section 1.3.8, demonstrating the advances of profile likelihood based reduction for non-trivial relations between different model components. In addition, a parameter reduction not proposed by the presented reduction algorithm is illustrated in Supplementary Section 1.3.9, resulting in a reduced model which is rejected by the likelihood-ratio test.

### Scenario 4: (−↕)

**Fig 5:**
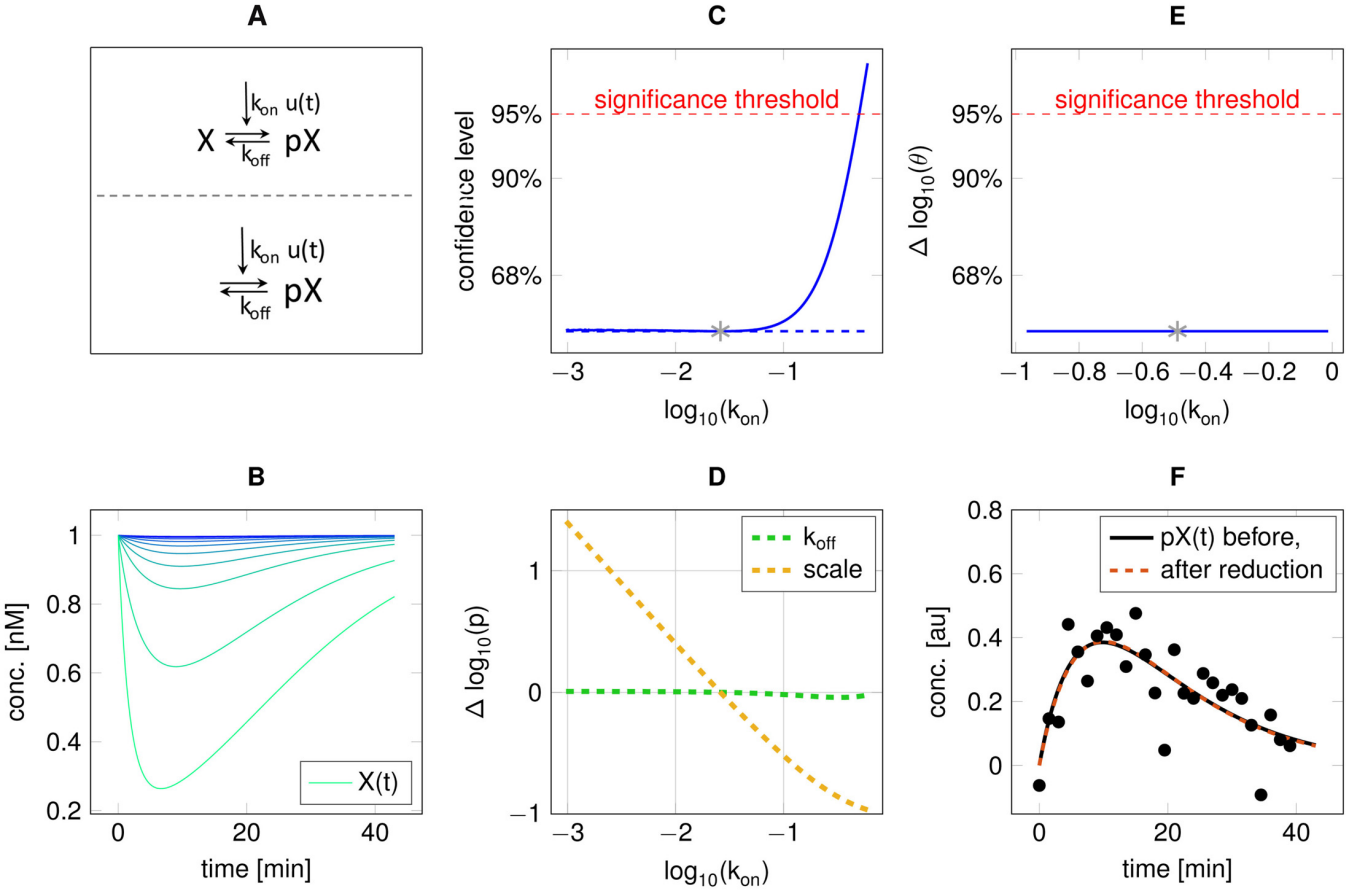
Model reduction of weakly activated signalling pathway (scenario 4). **A:** Model scheme before (upper) and after (lower) reduction. The difference is the omission of state *X*. B: *X*(*t*) for parameter sets along the profile likelihood of *k*_*on*_ (high to low values of *k*_*on*_ from bottom to top, i.e. cyan to blue). **C:** Parameter profile likelihood of *k*_*on*_, depicting a practical non-identifiability to small values. **D:** Relation of *scale* to *k*_*on*_. **E:** After model reduction, the parameter *k*_*on*_ is structurally non-identifiable and can be set to an arbitrary value. **F:** Comparison of model fits before and after reduction. Both curves overlap and cannot be statistically distinguished based on the data.

In the final toy example of a weakly activated signalling pathway, the reduction is based on the trajectories associated with the parameter profile likelihood. State *X* is simulated to be phosphorylated by an exponentially decaying input through *k*_*on*_, whereby the phosphorylated state *pX* can be deactivated with rate constant *k*_*off*_ (Fig 5A). In addition, *pX* is mapped to the observations by a scaling factor, i.e. *pX*_*obs*_ = *scale* ⋅ *pX*. Data is observed for *pX*(*t*) with standard deviation *σ* = 0.1 (Fig 5F). The parameter profile likelihood reveals that *k*_*on*_ (Fig 5C) is practically non-identifiable and the concomitant change of the parameter *scale* along the profile likelihood reveals that only their product is identifiable due to the linear relationship of log(*scale*) to log(*k*_*on*_) in Fig 5D. The reason that these are not structurally non-identifiable is revealed by plotting the trajectories associated with the parameter sets at each point of the profile likelihood (Fig 5B). As the initial level of *X* is fixed, the time course of its concentration level *X*(*t*) tends towards zero for large *k*_*on*_, i.e. *X* may become limiting (cyan trajectories in Fig 5B). If *X* is limiting, though, the shape of *pX*(*t*) is different from the weakly activated case, where the time course of *X*(*t*) converges to a nearly flat trajectory (blue trajectory in Fig 5B). These dynamics are associated with the flat direction of the parameter profile likelihood. Thus, the appropriate model reduction in this case is obtained by assuming that phosphorylation of *X* leads to a *negligible reduction* of the concentration of unphosphorylated *X*, which then serves as constant input for its phosphorylation. Thereby, the former practical non-identifiablility becomes a structural non-identifiability, which is depicted by the parameter profile likelihood of *k*_*on*_ after reduction (Fig 5E). Similar to Fig 5D, the parameter *scale* is coupled to *k*_*on*_ but as their relationship is now structural the model can be reduced by fixing one of them, e.g. *scale*, to an arbitrary value. After this reduction, the data is equally well described (Fig 5F).

### Flow-chart

**Fig 6:**
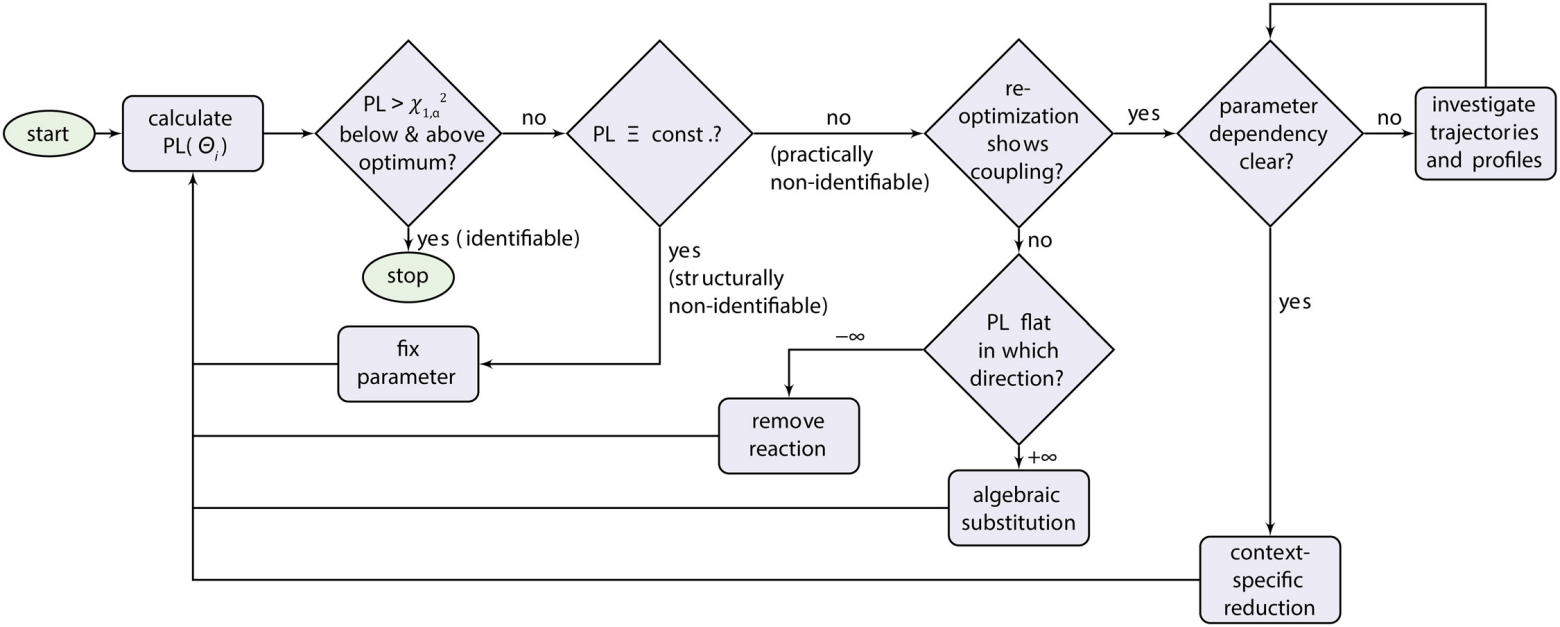
Typical flow-chart for model reduction based on the profile likelihood. The depicted steps are to be applied for each parameter individually, starting with the calculation of the respective profile likelihood. After detection, the procedure resolves non-identifiabilities by fixing parameters, removing reactions, performing algebraic substitutions, or context-specific reductions. The method terminates when all parameters of interest are identifiable.

A summary of the presented model reduction method is depicted in a flow-chart diagram in Fig 6. The proposed reduction steps are context-specific and should not be applied without checking their validity. Especially for scenarios 3 and 4, where coupling of parameters is present (↕), careful investigation of the parameter dependencies and their influence on the model quantities is crucial. On the contrary, this context-specific step provides additional insight, since relationships between parameters are revealed by driving the model towards its limits through the profile likelihood. These dependencies yield sub-systems of the original model and show the variability present within this sub-system. This modularisation identifies well-informed parts, and allows for reduction of uninformed parts.

### Model reduction of Reelin signalling cascade

In the following, the presented model reduction strategy will be carried out on a model of the early Reelin signalling cascade in order to demonstrate its applicability on real-world models of biochemical reactions. The Reelin signalling cascade is essential for the development of the mammalian brain by regulating the position of new-born neurons in the neocortex, hippocampus and cerebellum [32, 33]. Moreover, the pathway is involved in the modulation of synaptic plasticity, learning and memory in the adult brain, and defects in Reelin signalling are associated with neuropsychiatric diseases such as Alzheimer disease, schizophrenia, autism and epilepsy [34–36]. Although important aspects of Reelin signalling have been deciphered using classical biochemical approaches and mouse genetics, a superordinate view of the interaction of its components is needed [37].

**Fig 7:**
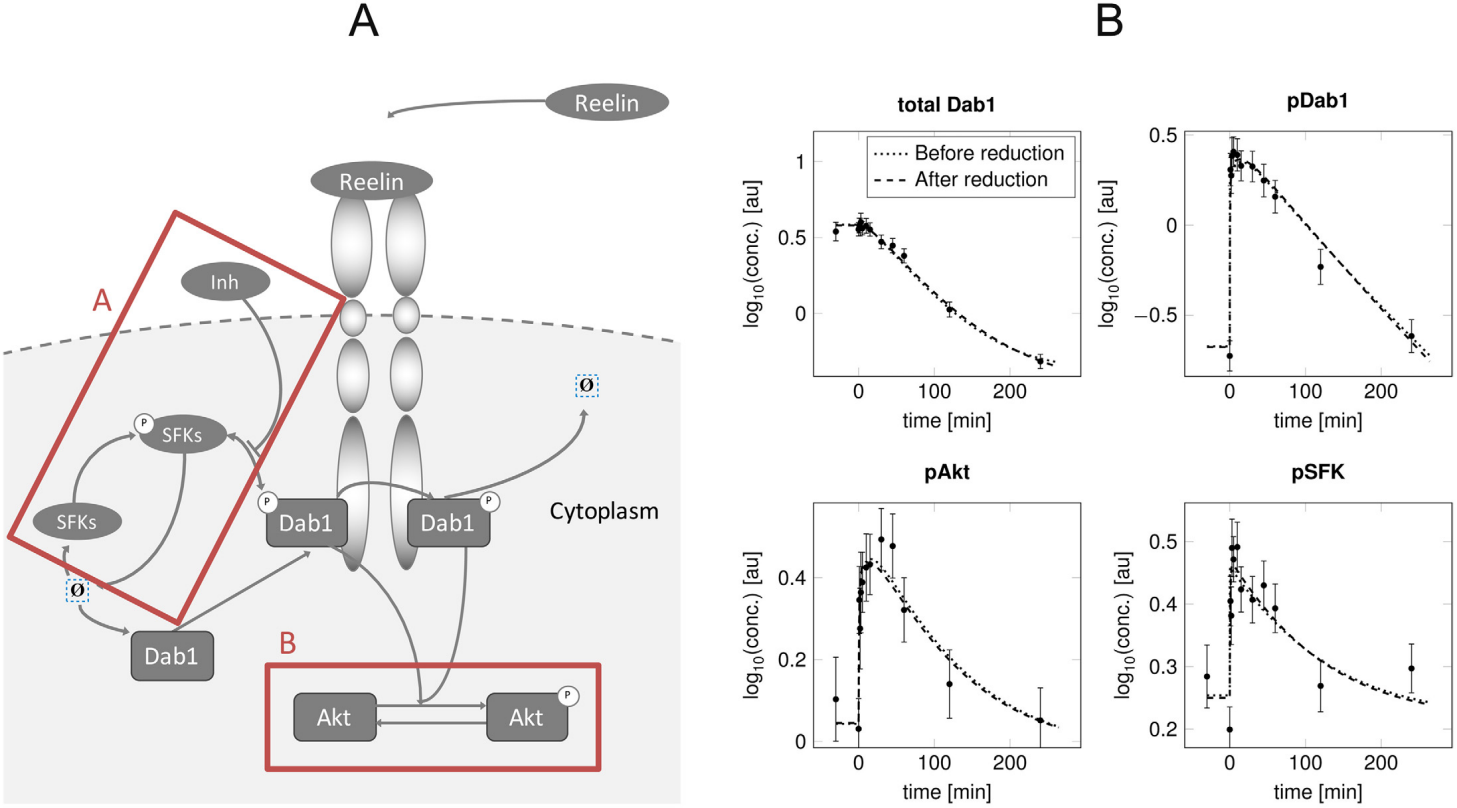
Reelin-induced signalling pathway. **A**: Scheme of the Reelin-induced signalling pathway. Sub modules that underwent model reduction are framed. **B**: Experimental data and model trajectories of the Reelin model. Data points and their measurement errors are shown on a log-scale for the measurements of total Dab1, phosphorylated Dab1, Akt and SFKs for time points between zero and 240 minutes. Dotted and dashed lines indicate the model response for the parameter set before and after model reduction, respectively.

Reelin exerts its function by binding to the lipoprotein receptors VLDLR and ApoER2, which induce tyrosine phosphorylation of the adaptor protein Dab1 through Src family kinases (SFKs) [38, 39]. As a feed-forward loop, phosphorylated Dab1 then trans-phosphorylates other Dab1 proteins bound to the receptor complex. In turn, Dab1 activates Akt that is, amongst others, involved in cell survival and migration (Fig 7A). The signalling is terminated by ubiquitination and degradation of Dab1. The proposed model reduction strategy is illustrated based on time-resolved data of total Dab1 and phosphorylated Dab1, SFKs and Akt, which were measured in cortical neurons after Reelin stimulation. In addition, an experiment applying a SFK inhibitor prior to Reelin stimulation was performed. Protein concentrations were measured in cell lysates of primary cortical neuron cultures by immunoblotting (see Supplementary Section 2). The measurements cover the first four hours after ligand stimulation, with dense measurements in the first 30 minutes (Fig 7B). Analytic steady state solutions, e.g. through basal phosphorylation, were determined for the initial concentration levels. The model consists of 12 states with initial concentrations implicitly given by steady state assumptions, 13 kinetic parameters, and 23 observational parameters. Taken together, the model therefore has 35 free parameters, which are fitted to 108 data-points. Computation of one parameter profile likelihood for this setting takes less than one minute on a standard laptop. Details about the data, model fits and differential equations before and after reduction are provided in Supplementary Section 2 and 3.

**Fig 8:**
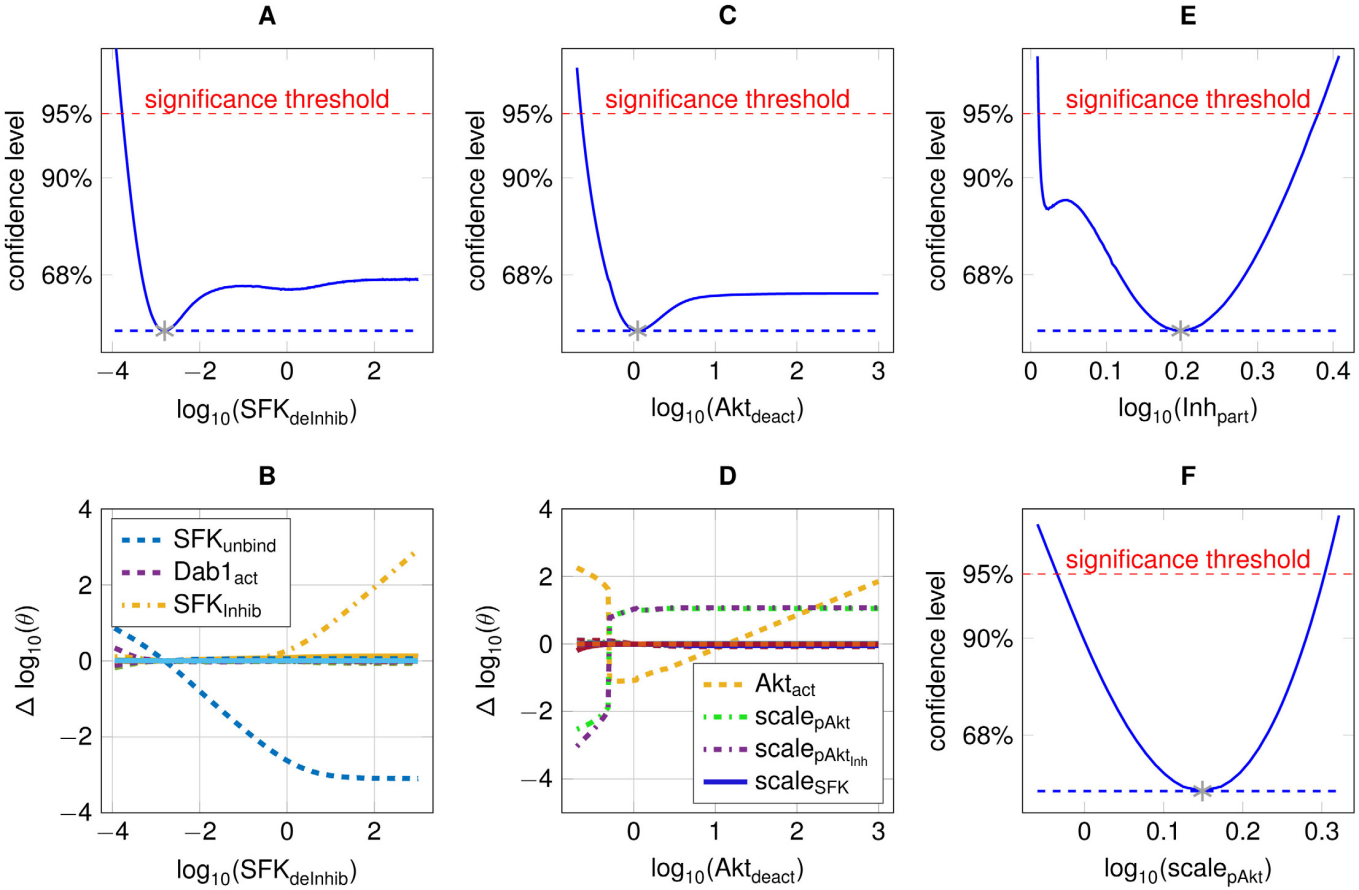
Summary of model reduction steps. **A**: The profile likelihood of the inhibitor release from SFKs. **B**: The re-optimised paths of the remaining parameters with respect to the profile of the inhibitor release. **C**: Parameter profile likelihood of the Akt deactivation. **D**: Coupling of the Akt activation, log(*Akt*_*act*_), to the Akt deactivation shown in panel C. **E**: Identifiable partitioning of SFKs with and without bound inhibitor. **F**: The scaling factor *scale*_*pAkt*_, which links the observations of pAkt to pDab1, is identifiable.

Considering the model parameters, two reductions can be conducted, which both correspond to scenario 3 (+↕). In Fig 7A, they are marked as red boxes named A and B. Regarding box A, the profile likelihood for the release of the SFK inhibitor, *SFK*_*deInhib*_, does not exceed the 95% threshold for large values (Fig 8A) showing a positive relation to its binding to the SFKs, *SFK*_*Inhib*_ (Fig 8B). This indicates that a steady state is reached instantly at every time point, which results in a consistent partitioning between SFKs with and without bound inhibitor. The factor *Inh*_*part*_, which determines the equilibrium concentration, is thereby identifiable (Fig 8E).

Further, the Akt deactivation *Akt*_*deact*_ shows a parameter profile that does not exceed the 95% threshold for large parameter values (Fig 8C), depicted by box B of Fig 7A. The Akt activation along the profile likelihood (Fig 8C) adapts to the Akt deactivation, as their ratio determines the steady state concentration of pAkt after Reelin stimulation (Fig 8D). A helpful fact in order to resolve this non-identifiability is that the time-course of pAkt will approach the time-course of the upstream protein Dab1 for large values of both Akt activation and deactivation. This result is not trivial, since scaling and offset linking both states to their respective observations are different. Replacing the Akt phosphorylation by a functional relation between pAkt and pDab1 resolves this non-identifiability. Thus, the pAkt dynamics follow the pDab1 dynamics, and can be mapped with an identifiable scaling factor *scale*_*pAkt*_ to the pAkt observations (Fig 8F). Fig 7B shows the insignificant impact of all model reduction steps on the dynamics. Statistical agreement of both the original and the reduced model is assessed by the likelihood-ratio test described in Eq (8), taking the difference in degrees of freedom between both models into account.

## Discussion and Conclusion

Traditionally, mathematical models are small since they phenomenologically describe processes like biochemical interactions at a simplified level. In such a setting, one question is whether an initially chosen level of abstraction turns out to be too stringent demanding for to model enlargement by additional effects. In contrast to this traditional situation, mathematical models in systems biology are usually mechanistic, i.e. all relevant processes in living cells have their counterparts in the model. Therefore, the models typically start out large and the adequate level of complexity has to be checked in both directions, i.e. on the one hand it has to be tested whether all essential processes and interactions are included and on the other hand model reductions can be performed. Following such a combined strategy offers the possibility to obtain a comprehensive model, which is tailored to include only the required components.

The model reduction methodology presented in this manuscript is purely data-based, yet can take prior knowledge into account. We use the profile likelihood, which is a continuous representation of the likelihood ratio statistic, to suggest a model reduction strategy that iteratively eliminates practically non-identifiable parameters. It considers the distribution and magnitude of data noise in a statistically valid manner and is robust against observation noise. Investigating the model fit along the profile likelihood and considering parameter coupling provides educated guesses on where the model may be amenable to reduction. Increasing levels of data noise would affect the method in a way that more simulation trajectories would be able to describe the data within the statistical boundaries set by the *χ*^2^ function.

Our suggested approach permits simplifications, which would not be rejected by the likelihood-ratio test. However, because only experimentally observed model components and incorporated prior knowledge enter the likelihood ratio, such model simplifications face the risk that unobserved but biologically relevant parts are removed which would manifest in biased predictions. Unfortunately, significance considerations are not applicable to this issue since statistical tests only permit of model simplifications but not of generalisations. However, the risk of removing relevant parts can be diminished by restricting to biologically meaningful simplifications and by performing validation experiments. Except for this important aspect, the presented model reduction strategy can be performed iteratively until an identifiable model is obtained which typically enables precise predictions with small confidence intervals that facilitate experimental validation or falsification of the current model structure. Moreover, an identifiable model permits summarising the experimental outcomes to estimated parameters and confidence intervals of finite size which is an important requirement for transferring a model to new applications. Additionally, identifiable models require less demanding computations at several levels. Optimisation of the parameters for instance is less hampered by ill-conditioned step size control. Moreover, the performance of ODE integration deteriorates less frequently because rate constants are restricted to meaningful ranges and the ODEs tend to be non-stiff.

In this study, we do not consider the specific order in which the model reduction steps are performed. This issue occurs in any model reduction and model discrimination problem and is usually approached by either completely searching all admissible cases or by stepwise forward or backward selection or combinations thereof. As an example, [15] consider the order of model reduction steps by comparing it to the traveling salesman problem. Although not shown here, established approaches targeting this issue can be readily combined with the presented model reduction steps.

The profile likelihood has become a well-established method for the analysis of parameter uncertainty. Its calculation is often routinely performed in systems biology applications. In such cases, our model reduction analysis can be performed without additional effort. In addition, the computation of parameter profiles can be performed in parallel on a multi-core processor or by means of distributed computing.

To illustrate our profile likelihood-based model reduction strategy, we focused on examples with general impact covering a broad range of model redundancies. However, these examples are not exhaustive and additional reductions may occur in special cases. If such context-specific model reductions are required, investigation of model predictions, e.g. trajectories of the internal states *x*(*t*), for parameter sets sampled along the profile likelihood is often helpful.

Thereby, the presented method reveals features which are crucial for model predictions while the simplifications can be traced back to their mechanistic origin. Thus, the approach iteratively builds up confidence in the model, and constitutes a helpful tool for improving understanding, interpretability, and usefulness of systems biology models.

## Supporting Information

S1 File Implementations and analyses of toy models and further documentation on the Reelin pathway model. (PDF)
