# Supplementary material for: Driving the Model to Its Limit: Profile Likelihood Based Model Reduction
Source: PLoS One. 2016 Sep 2;11(9):e0162366. doi: 10.1371/journal.pone.0162366 (PMC5010240; doi:10.1371/journal.pone.0162366)
Supplement: S1 File — (PDF) [file pone.0162366.s001.pdf]

Driving the model to its limit: profile likelihood based model reduction

Tim Maiwald, Helge Hass, Bernhard Steiert, Joep Vanlier, Raphael Engesser, Andreas Raue, Friederike Kipkeew, Hans H. Bock, Daniel Kaschek, Clemens Kreutz, and Jens Timmer

Contents

1 Examples implemented in dMod/cOde 3
1.1 Scenario 1: (+|) 3
1.1.1 Model scheme, equations and parameters 3
1.1.2 Model definition 3
1.1.3 Simulation of data 4
1.1.4 Model reduction analysis 5
1.1.5 Conclusion 6
1.2 Scenario 2: (−|) 7
1.2.1 Model scheme, equations and parameters 7
1.2.2 Model definition 7
1.2.3 Simulation of data 8
1.2.4 Model reduction analysis 9
1.2.5 Conclusion 10
1.3 Scenario 3 (+ $\updownarrow$) 11
1.3.1 Model scheme, equations and parameters 11
1.3.2 Model definition 11
1.3.3 Simulation of data 13
1.3.4 Model reduction analysis 13
1.3.5 Parameter paths and model prediction along the profile 15
1.3.6 New observable: pC/pB 17
1.3.7 Conclusion 18
1.3.8 Flux-based reduction 18
1.3.9 Deletion of identifiable parameters 18
1.4 Scenario 4: (− $\updownarrow$) 19
1.4.1 Model scheme, equations and parameters 19
1.4.2 Model definition 20
1.4.3 Simulation of Data 21
1.4.4 Model reduction analysis 22
1.4.5 Conclusion 24

2 **Model of reelin signalling pathway** 25
2.1 Functioning of Reelin . . . 25
2.2 Preparation and treatment of primary cortical neuron cultures . . . 25
2.3 Western blotting . . . 26
2.4 Mathematical modelling . . . 26
2.5 Dynamic parameters . . . 27
2.6 Model fit and plots . . . 29
2.7 Experimental condition with SFK inhibition . . . 31
2.8 Parameter profiles . . . 32

3 **Model after full reduction** 35
3.1 Dynamic parameters . . . 35

1 Examples implemented in dMod/cOde

Get the packages from GitHub:

```
devtools::install_github("dkaschek/cOde")
devtools::install_github("dkaschek/dMod")
```

1.1 Scenario 1: (+|)

1.1.1 Model scheme, equations and parameters

Reaction scheme:

$$X \xrightarrow{k_1} pX \xrightarrow{k_2} ppX$$

Equations:

$$\begin{aligned} \dot{X} &= -k_1 \cdot X \\ p\dot{X} &= k_1 \cdot X - k_2 \cdot pX \\ pp\dot{X} &= k_2 \cdot pX \end{aligned}$$

Parameters:

$$\begin{aligned} X(0) &= 1 \\ pX(0) = ppX(0) &= 0 \\ k_1 &= 10^5 \\ k_2 &= 10^{-1} \end{aligned}$$

1.1.2 Model definition

```
# Load libraries
library(deSolve)
library(parallel)
library(dMod)
```

```
# Generate reaction network
f <- NULL
f <- addReaction(f, "X", "pX", "k1 * X")
f <- addReaction(f, "pX", "ppX", "k2 * pX")

# Generate the model C files, compile them and return a list with func and extended.
model0 <- odemodel(f, compile = TRUE, modelname = "odefn1")

# Define inner parameters (parameters occurring in the equations except forcings)
innerpars <- getParameters(model0)

# Define additional parameter constraints, e.g. initial states
constraints <- c(
```

```r
  X   = "1",
  pX  = "0",
  ppX = "0"
)

# Box constraint parameterization
box <- function(p, upperhalf = 5e5)
  paste0("(", upperhalf, "+", upperhalf, "*tanh(", p, "))")
unbox <- function(p, upperhalf = 5e5) atanh(p/upperhalf-1)

# Build up a parameter transformation (constraints, log-transform, etc.)
# Start with the identity
trafo <- structure(innerpars, names = innerpars)
# Then employ the other parameter constraints
trafo <- replaceSymbols(names(constraints), constraints, trafo)
# Then do a box-transform of all parameters
trafo <- replaceSymbols(innerpars, box(innerpars), trafo)
# Get names of new parameters
outerpars <- getSymbols(trafo)

# Generate parameter transformation function
p0 <- P(trafo, condition = "cond1")

# Generate prediction function
x0 <- Xs(model0)

# Generate prediction function with parameter transformation
y <- x0*p0
```

1.1.3 Simulation of data

```r
# Use the following parameters
pouter <- c(k1 = unbox(1e5),
            k2 = unbox(0.1))

# Constant noise level and equidistant time points
noise <- 0.1
timesD <- seq(0, 60, by = 3)
set.seed(4)

# Predict model response and add noise
prediction <- y(timesD, pouter)
values.C <- prediction$cond1[, "ppX"]
noise.C <- rnorm(length(values.C), 0, noise)

data <- datalist(
  cond1 = data.frame(name = "ppX",
                     time = timesD,
                     value = values.C + noise.C,
                     sigma = noise)
)
```

1.1.4 Model reduction analysis

```
# Initalize parameters
fixed <- NULL
pinit <- pouter[setdiff(outerpars, names(fixed))]
times <- seq(0, 60, .5)

# Fit parameters and plot prediction and data
myfit <- trust(normL2(data, y), pinit, rinit = 1, rmax = 10, fixed = fixed)
plot(y(times, myfit$argument, fixed = fixed), data)
```

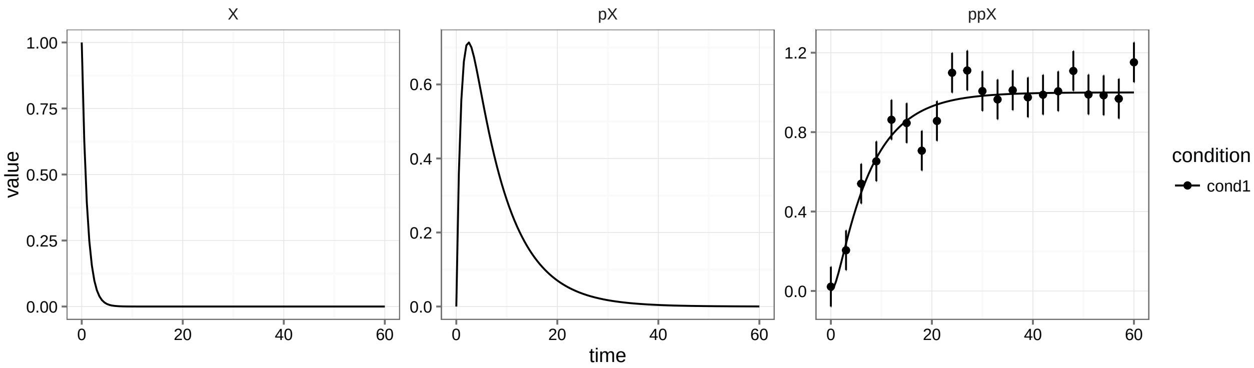

Figure 1: Model trajectories and data.

```
# Compute profile
bestfit <- c(myfit$argument, fixed)
prior <- bestfit
myprof <- profile(normL2(data, y), pars = bestfit, whichPar = "k1", limit = c(-10, 10),
                  stepControl = list(atol = 1e-2, rtol = 1e-2),
                  method = "optimize")

# Plot profile likelihood
plotProfile(myprof)
```

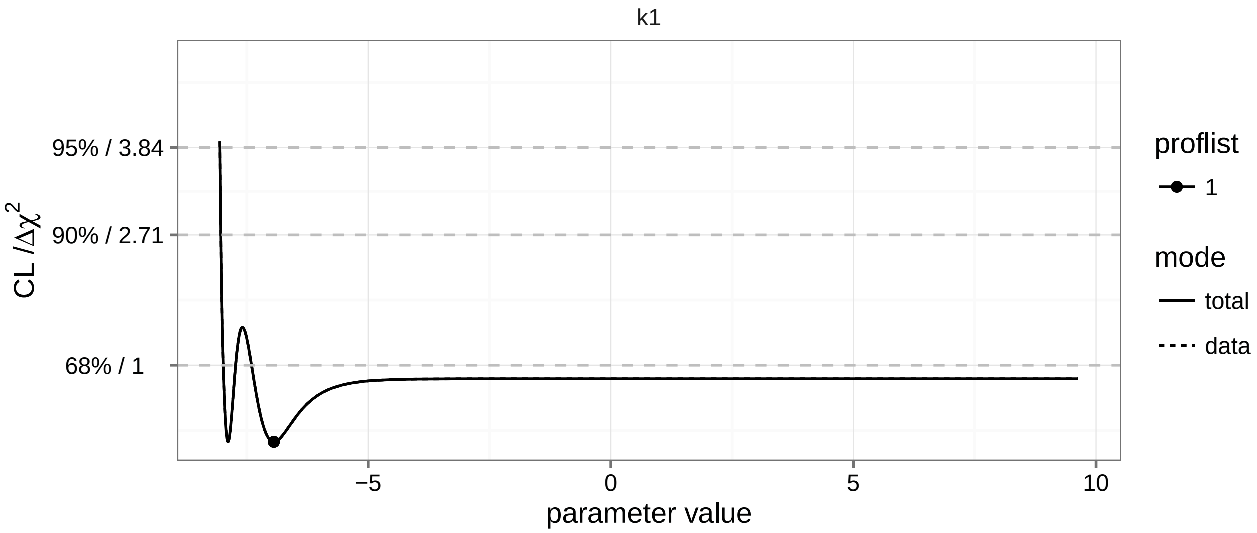

Figure 2: Profile likelihood of parameter k1.

```
# Plot associated parameter paths
plotPaths(myprof) + ylim(c(NA, 3))
```

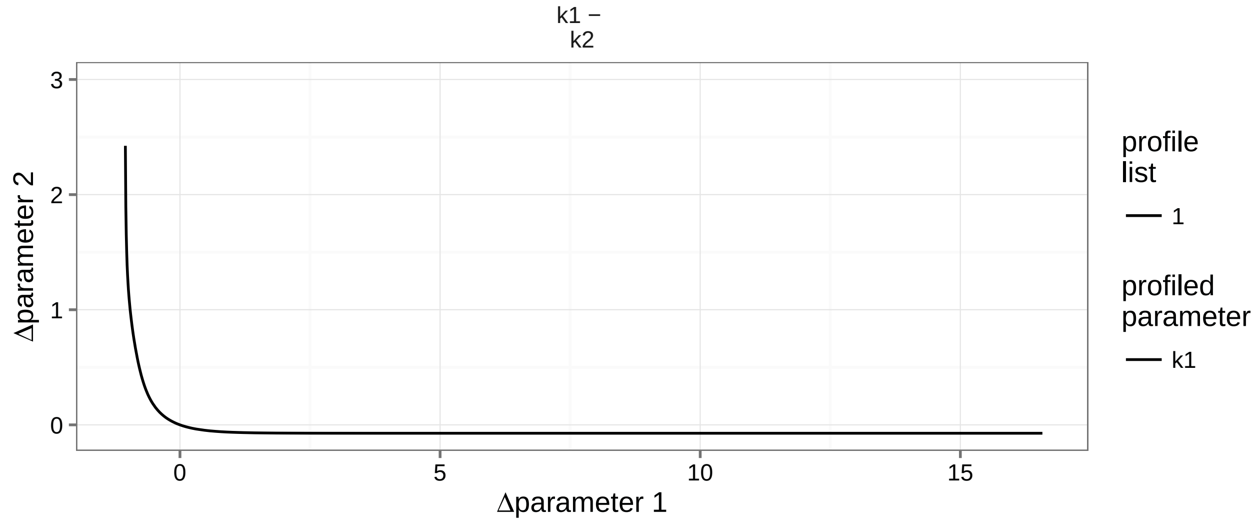

Figure 3: Associated parameter paths.

1.1.5 Conclusion

In the limit $k_1 \rightarrow \infty$ the parameter k_2 converges to a finite value. Hence, the first reaction can be arbitrarily fast, meaning the system is (almost) equivalently expressed by the model

$$X \xrightarrow{k} ppX$$

1.2 Scenario 2: (—|)

1.2.1 Model scheme, equations and parameters

Reaction scheme:

$$X \underset{k_4+k_5 ppX}{\overset{k_1}{\rightleftharpoons}} pX \xrightarrow{k_2} ppX$$

Equations:

$$\begin{aligned}
\dot{X} &= -k_1 \cdot X + k_4 \cdot pX + k_5 \cdot pX \cdot ppX \\
\dot{pX} &= k_1 \cdot X - k_4 \cdot pX - k_5 \cdot pX \cdot ppX - k_2 pX \\
\dot{ppX} &= k_2 \cdot pX
\end{aligned}$$

Parameters:

$$\begin{aligned}
X(0) &= 1 \\
pX(0) = ppX(0) &= 0 \\
k_1 &= 10^{-1} \\
k_2 &= 10^{-3} \\
k_4 &= 10^{-3} \\
k_5 &= 2
\end{aligned}$$

1.2.2 Model definition

```
# Load libraries
library(deSolve)
library(parallel)
library(dMod)
```

```
# Generate reaction network
f <- NULL
f <- addReaction(f, "X", "pX", "k1 * X")
f <- addReaction(f, "pX", "ppX", "k2 * pX")
f <- addReaction(f, "pX", "X", "k4 * pX")
f <- addReaction(f, "pX", "X", "k5 * ppX * pX")

# Generate the model C files, compile them and return a list with func and extended.
model0 <- odemodel(f, compile = TRUE, modelname = "odefn2")

# Define inner parameters (parameters occurring in the equations except forcings)
innerpars <- getParameters(model0)

# Define additional parameter constraints, e.g. initial states
constraints <- c(
  X = "1",
  pX = "0",
  ppX = "0"
)
```

```r
# Box constraint parameterization
box <- function(p, upperhalf = 5e5)
  paste0("(", upperhalf, "+", upperhalf, "*tanh(", p, "))")
unbox <- function(p, upperhalf = 5e5) atanh(p/upperhalf - 1)

# Build up a parameter transformation (constraints, log-transform, etc.)
# Start with the identity
trafo <- structure(innerpars, names = innerpars)
# Then employ the other parameter constraints
trafo <- replaceSymbols(names(constraints), constraints, trafo)
# Then do a box-transform of all parameters
trafo <- replaceSymbols(innerpars, box(innerpars), trafo)
# Get names of new parameters
outerpars <- getSymbols(trafo)

# Generate parameter transformation function
p0 <- P(trafo, condition = "cond1")

# Generate prediction function
x0 <- Xs(model0)

# Generate prediction function with parameter transformation
y <- x0*p0
```

1.2.3 Simulation of data

```r
# Use the following parameters
pouter <- unbox(c(k1 = 1e-1,
                  k2 = 1e-3,
                  k4 = 1e-3,
                  k5 = 2))

# Constant noise level and equidistant time points
noise <- 0.02
timesD <- seq(0, 100, by = 3)
set.seed(3)

# Predict model response and add noise
prediction <- y(timesD, pouter)
values.A <- prediction$cond1[, c("X", "pX")]
noise.A <- rnorm(length(values.A), 0, noise)



data <- datalist(
  cond1 = data.frame(name = rep(c("X", "pX"), each = nrow(values.A)),
                     time = timesD,
                     value = as.numeric(values.A + noise.A),
                     sigma = noise)
)
```

1.2.4 Model reduction analysis

```r
# Initalize parameters
fixed <- NULL
pinit <- pouter[setdiff(outerpars, names(fixed))]
times <- 0:100

# Fit parameters and plot prediction and data
myfit <- trust(normL2(data, y), pinit, rinit = 1, rmax = 10, fixed = fixed)
plot(y(times, myfit$argument, fixed = fixed), data)
```

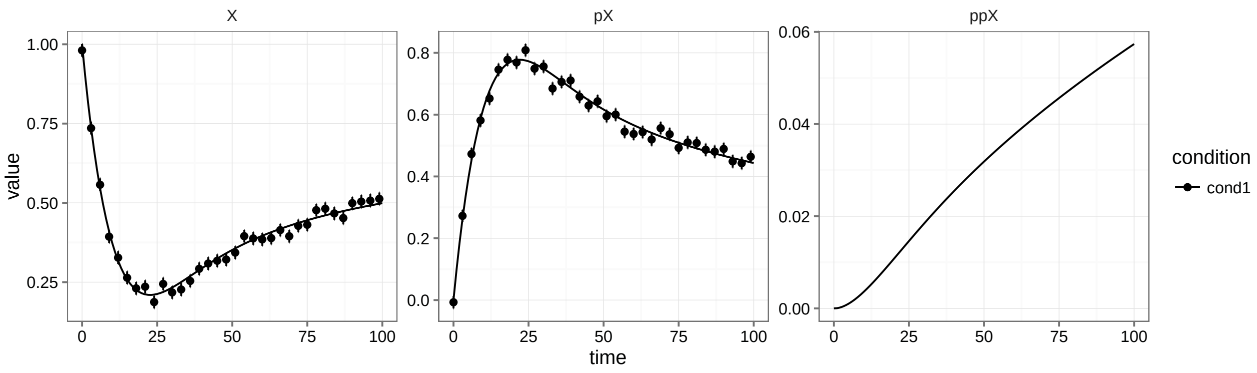

Figure 4: Model trajectories and data.

```r
# Compute profile
bestfit <- c(myfit$argument, fixed)
prior <- bestfit
myprof <- rbind(
  profile(normL2(data, y), pars = bestfit, whichPar = "k4", limit = c(-10, 20),
          stepControl = list(atol = 1e-2, rtol = 1e-2, limit = Inf, max = .5),
          method = "optimize"),
  profile(normL2(data, y), pars = bestfit, whichPar = "k5", limit = c(-10, 20),
          stepControl = list(stepsize = 1e-4, atol = 1e-2, rtol = 1e-2),
          method = "optimize")
)

# Plot profile likelihood
plotProfile(myprof)
```

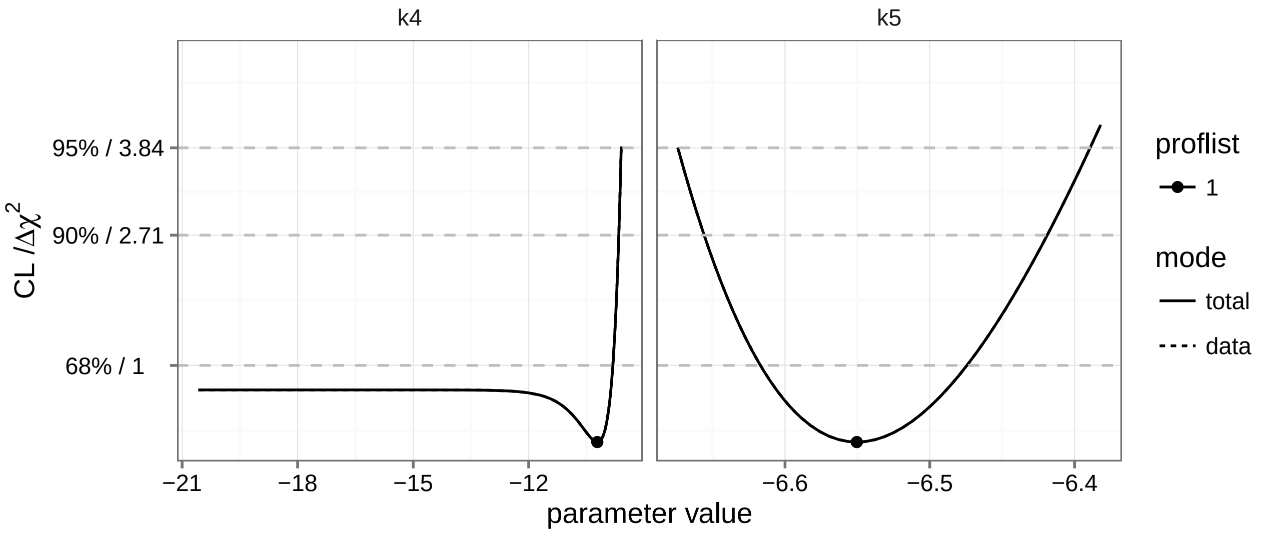

Figure 5: Profile likelihoods of parameters k4 and k5.

```r
# Plot associated parameter paths
plotPaths(myprof, whichPar = "k4")
```

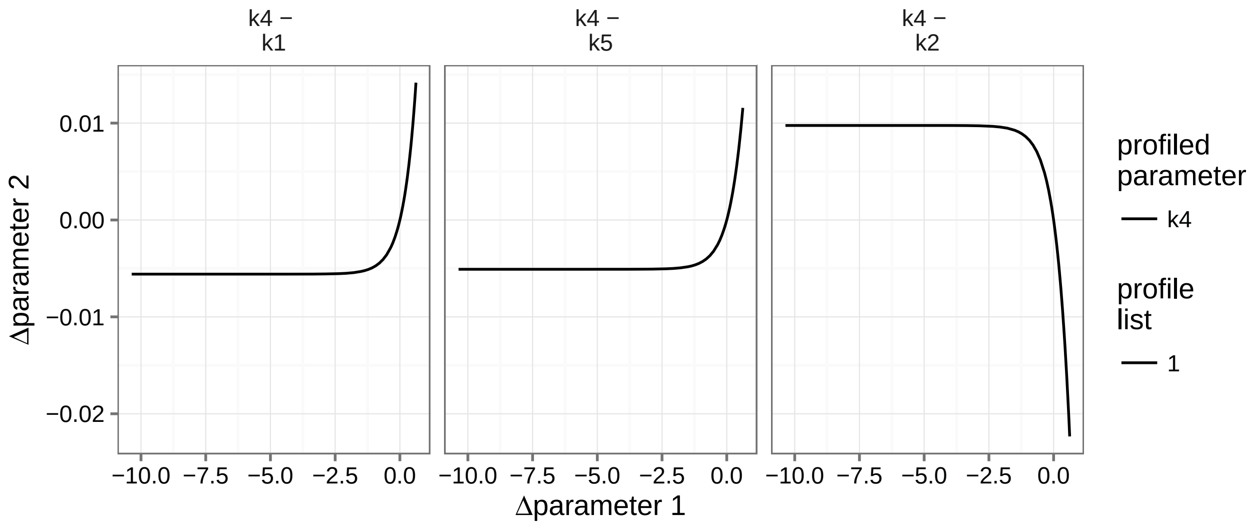

Figure 6: Associated parameter paths.

1.2.5 Conclusion

In the limit $k_4 \to -\infty$ no other parameter couples k_4. Therefore, the reaction $pA \xrightarrow{k_4} A$ can be removed from the networ without changing the fit. The reduced model reads:

$$X \xrightarrow{k_1} pX \xrightarrow{k_2} ppX$$

$$pX \xrightarrow{k_5 \cdot ppX} X$$

1.3 Scenario 3 ($+\updownarrow$)

1.3.1 Model scheme, equations and parameters

Reaction scheme:

$$\emptyset \xrightarrow{\text{X\_prod}} X \underset{\text{pX\_deact}}{\overset{\text{X\_act+X\_act\_L}\cdot\text{L}}{\rightleftharpoons}} pX \xrightarrow{\text{X\_degrad}} \emptyset$$

$$Y \underset{\text{pY\_deact}}{\overset{\text{Y\_act}\cdot\text{pX}}{\rightleftharpoons}} pY$$

$$Z \underset{\text{pZ\_deact}}{\overset{\text{Z\_act}\cdot\text{pY}}{\rightleftharpoons}} pZ$$

Equations:

$$\begin{aligned}
\dot{X} &= -k_1 \cdot X + k_4 \cdot pX + k_5 \cdot pX \cdot ppX \\
\dot{pX} &= k_1 \cdot X - k_4 \cdot pX - k_5 \cdot pX \cdot ppX - k_2 pX \\
\dot{ppX} &= k_2 \cdot pX
\end{aligned}$$

Observation:

$$\begin{aligned}
pX_au &= scale_X \cdot pX \\
pY_au &= scale_Y \cdot pY \\
pZ_au &= 100 \cdot pZ
\end{aligned}$$

Parameters:

$$\begin{aligned}
L = X(0) = Y(0) = Z(0) &= 1 \\
pX(0) = pY(0) = pZ(0) &= 0 \\
X_prod &= e^{-4} \\
X_act &= e^{-1} \\
X_act_L &= e^{-1} \\
X_deact &= e^{-9} \\
X_degrad &= e^{-0.3} \\
Y_act &= e^{-6} \\
pY_deact &= e^{-3} \\
Z_act &= e^{2} \\
pZ_deact &= e^{1} \\
scale_X &= e^{1.7} \\
scale_Y &= e^{5}
\end{aligned}$$

1.3.2 Model definition

```
# Load libraries
library(deSolve)
library(parallel)
library(dMod)
```

```r
# Generate reaction network
f <- NULL
f <- addReaction(f, "", "X", "X_prod")
f <- addReaction(f, "X", "pX", "X_act * X")
f <- addReaction(f, "X", "pX", "X_act_L * X * L")
f <- addReaction(f, "pX", "X", "X_deact * pX")
f <- addReaction(f, "pX", "", "X_degrad * pX")
f <- addReaction(f, "Y", "pY", "Y_act * Y * pX")
f <- addReaction(f, "pY", "Y", "pY_deact * pY")
f <- addReaction(f, "Z", "pZ", "Z_act * Z * pY")
f <- addReaction(f, "pZ", "Z", "pZ_deact * pZ")


# Define new observables based on ODE states
observables <- c(
  pX_au = "scale_X * pX",
  pY_au = "scale_Y * pY",
  pZ_au = "100 * pZ"
)

# Generate observation function
g <- Y(observables, f, compile = TRUE, modelname = "obsfn3")

# Generate the model C files, compile them and return a list with func and extended.
model0 <- odemodel(f, compile = TRUE, modelname = "odefn3")

# Define inner parameters (parameters occurring in the equations except forcings)
innerpars <- union(getParameters(model0), getParameters(g))

constraints <- resolveRecurrence(c(
  L = "1",
  pX = "0",
  pY = "0",
  pZ = "0",
  X = "1",
  Y = "1",
  Z = "1"
))

# Build up a parameter transformation (constraints, log-transform, etc.)
# Start with the identity
trafo <- structure(innerpars, names = innerpars)
# Replace constraints
trafo <- replaceSymbols(names(constraints), constraints, trafo)
# Then do a log-transform of all parameters (if defined as positive numbers)
trafo <- replaceSymbols(innerpars, paste0("exp(log", innerpars, ")"), trafo)
# Get names of new parameters
outerpars <- getSymbols(trafo)

# Generate parameter transformation function
p0 <- P(trafo, condition = "cond1")

# Generate prediction function
```

```r
x0 <- Xs(model0)

# Generate prediction function with obervation and parameter transformation
y <- g*x0*p0
```

1.3.3 Simulation of data

```r
# Use the following parameters
pouter <- c(logX_prod = -4,
            logX_act = -1,
            logX_act_L = -1,
            logX_deact = -9,
            logX_degrad = -0.3,
            logY_act = -6,
            logpY_deact = -3,
            logZ_act = 2,
            logpZ_deact = 1,
            logscale_X = 1.7,
            logscale_Y = 5)
# Constant noise level and equidistant time points
noise <- 0.05
timesD <- c(0, 2, 4, 7, seq(10, 100, by = 5))
set.seed(3)

# Predict model response and add noise
prediction <- y(timesD, pouter)
values.A <- prediction$cond1[, c("pY_au", "pZ_au")]
noise.A <- rnorm(length(values.A), 0, noise)



data <- datalist(
  cond1 = data.frame(name = rep(c("pY_au", "pZ_au"), each = nrow(values.A)),
                     time = timesD,
                     value = as.numeric(values.A + noise.A),
                     sigma = noise)
)
```

1.3.4 Model reduction analysis

```r
# Initalize parameters
times <- seq(0, 10, by = 0.1)^2
fixed <- NULL
#pouter <- structure(rep(1, length(outerpars)), names = outerpars)
pinit <- pouter[setdiff(outerpars, names(fixed))]

# Fit parameters and plot prediction and data
myfit <- trust(normL2(data, y), pinit, rinit = 1, rmax = 10, fixed = fixed, iterlim = 1e3)
plot(y(times, myfit$argument, fixed = fixed), data)
```

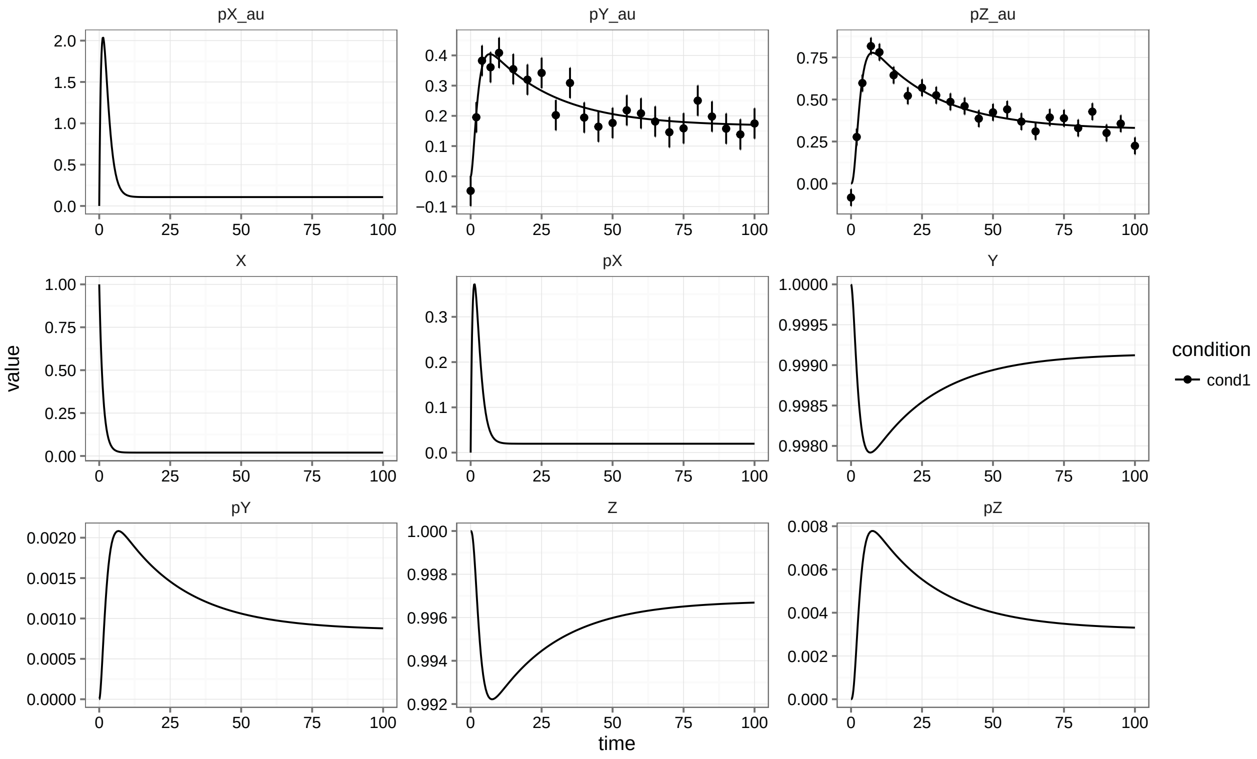

Figure 7: Model trajectories and data.

```r
# Compute profile
bestfit <- c(myfit$argument, fixed)
myprof <- profile(normL2(data, y), pars = bestfit, whichPar = "logpZ_deact", limit = c(-10, 10),
                  method = "optimize", stepControl = list(max = 1))
# Plot profile likelihood
plotProfile(myprof)
```

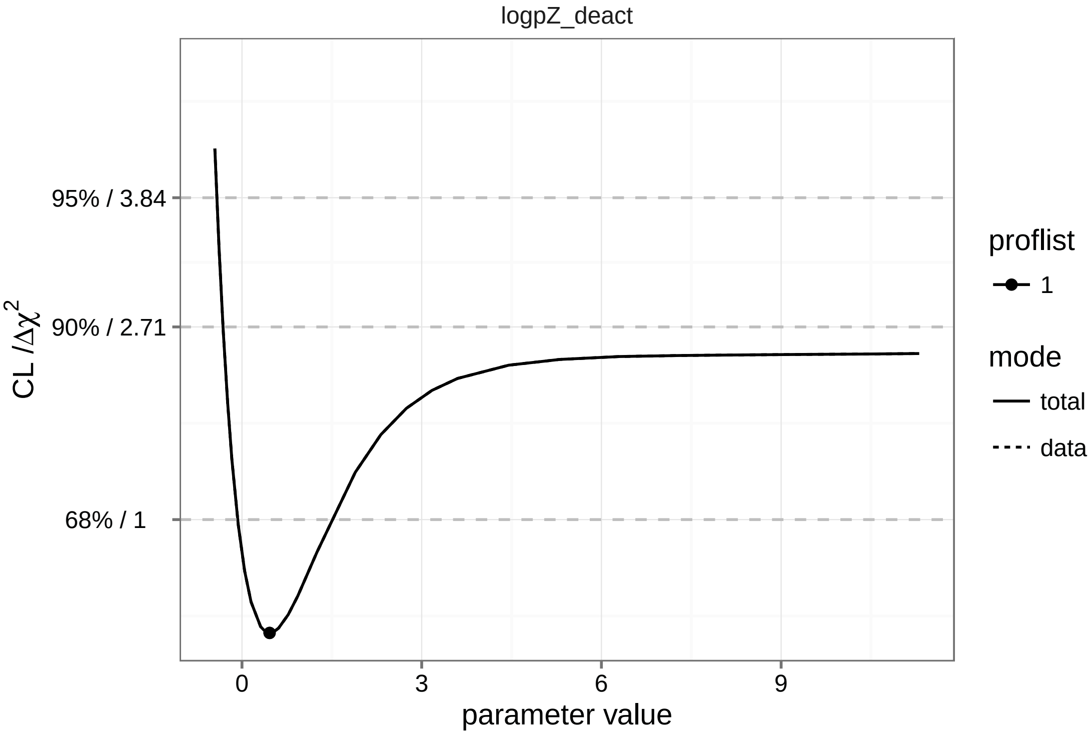

Figure 8: Profile likelihood of parameter pZ deact.

1.3.5 Parameter paths and model prediction along the profile

```
# Plot associated parameter paths
plotPaths(myprof)
```

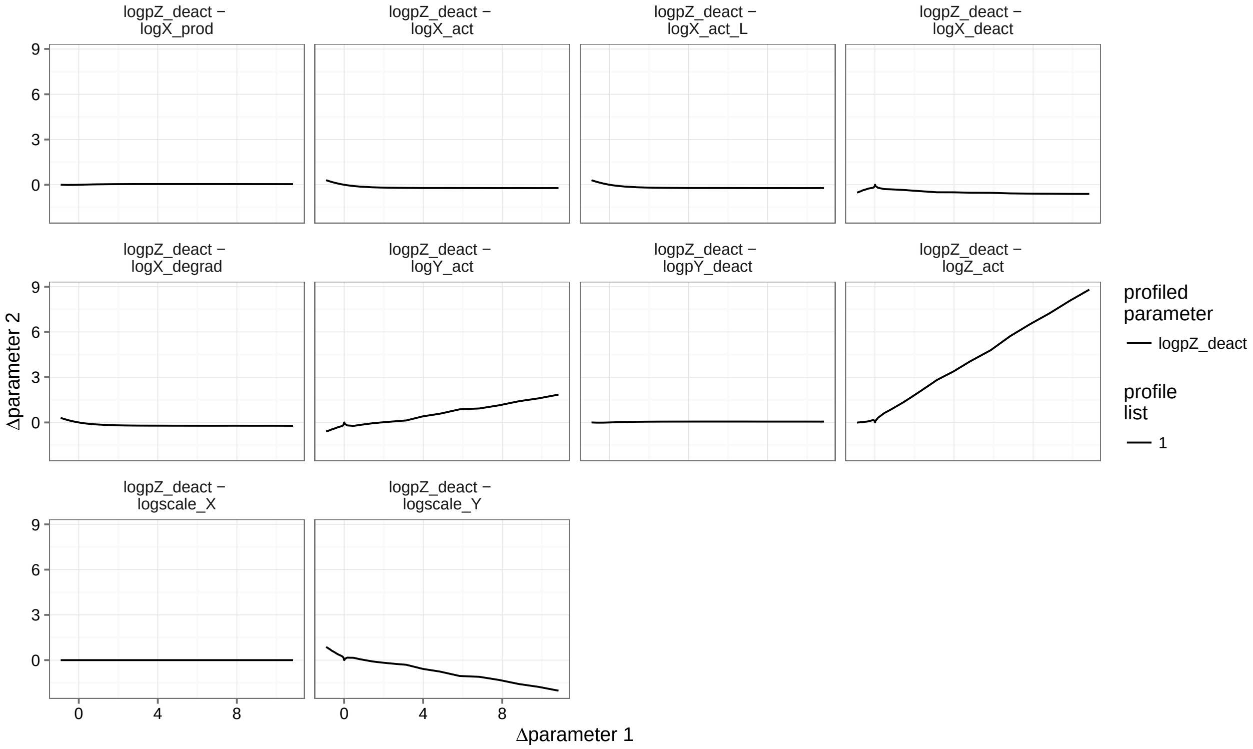

Figure 9: Associated parameter paths.

```
# Plot model prediction for right branch of the profile
plotArray(subset(myprof, constraint > 0), y, times, data) + scale_color_continuous(name = "objective\nva
```

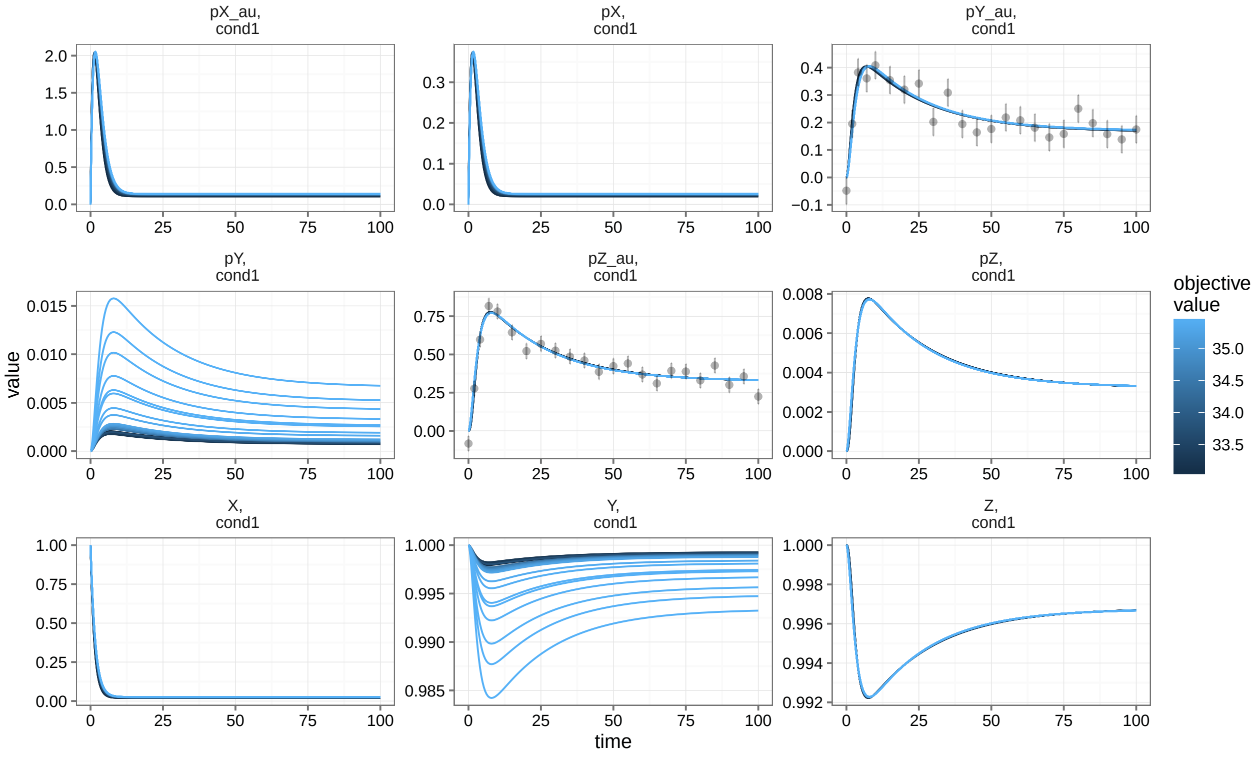

Figure 10: Model predictions for right branch of the profile.

1.3.6 New observable: pC/pB

```
g2 <- Y(c(ratio_pZ_pY = "pZ/pY"), f, attach.input = FALSE)

plotArray(subset(myprof, constraint > 0), g2*x0*p0, times[times <= 10]) +
  scale_color_continuous(name = "objective\nvalue")
```

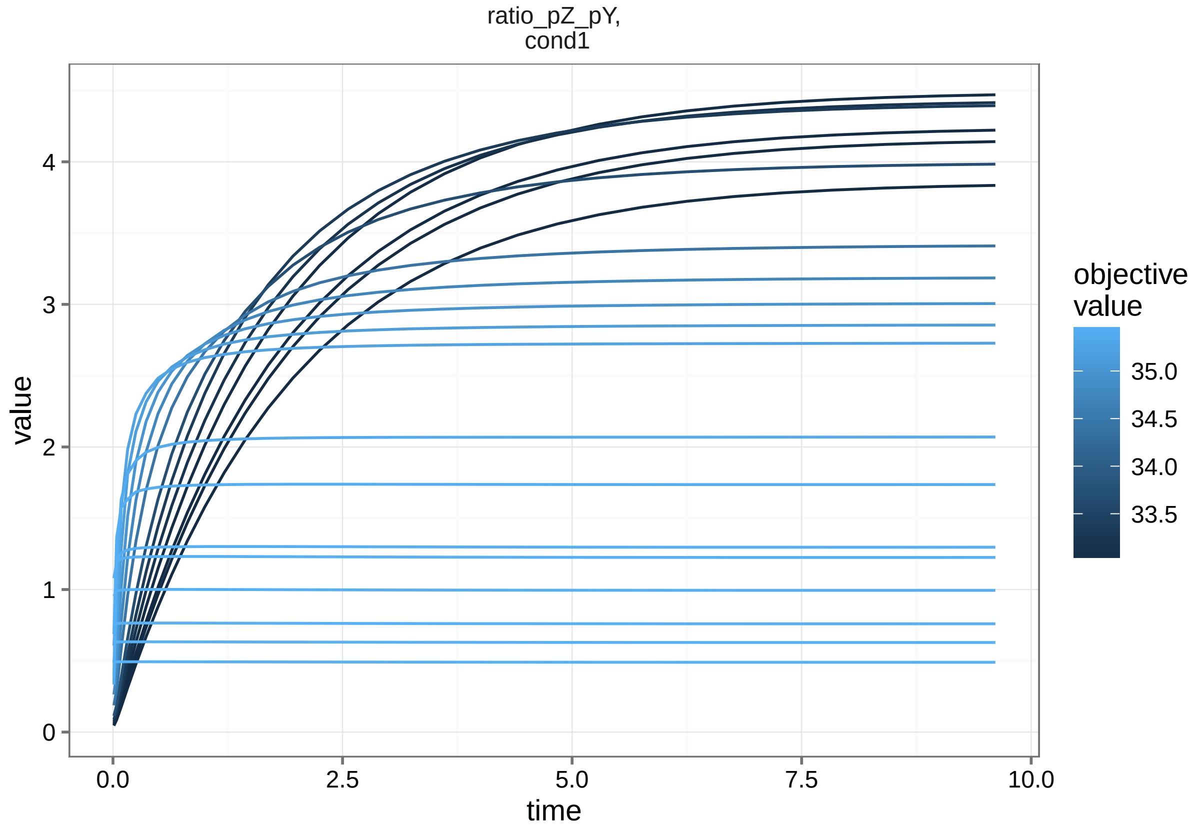

Figure 11: Model trajectories of the ratio pZ/pY.

1.3.7 Conclusion

In the limit $Z_{deact} \to \infty$, the parameters Z_{act} and initial value Z couple to Z_{deact} and tend to infinity, too. In this limit, the quotient $\frac{pY(t)}{pZ(t)} \to \mathtt{const}(t)$ tends to a constant. The reduced model does not contain Z or pZ any more but is fully described by the dynamics of Y via $pZ = \alpha pY$.

1.3.8 Flux-based reduction

For this example, the presented model reduction via profile likelihood is compared to a reduction based on negligible fluxes. Therefore, the six fluxes of the model, i.e. in- and outflow of states X, Y and Z are analyzed. In contrast to the practical non-identifiability and relation between k_Z and $k_{d,Z}$, the fluxes corresponding to the states X, Y and Z are on the same scale, respectively (Fig. 12). Thus, model reduction based on negligible fluxes does not reveal any possible model reduction. It results solely from the relationship between both parameters and a non-trivial relation of state Z to the upstream component Y, independent of the internal flux of the model.

1.3.9 Deletion of identifiable parameters

In the presented model reduction method, parameters prone for reduction are proposed based on their non-identifiability. In contrast, reduction of identifiable parameters should lead to significant decline of the likelihood and result in a worse description of the given measurements. To demonstrate the effect of a non-proposed model reduction, the parameter $\mathtt{pY\_deact}$ is set to zero instead of setting a functional

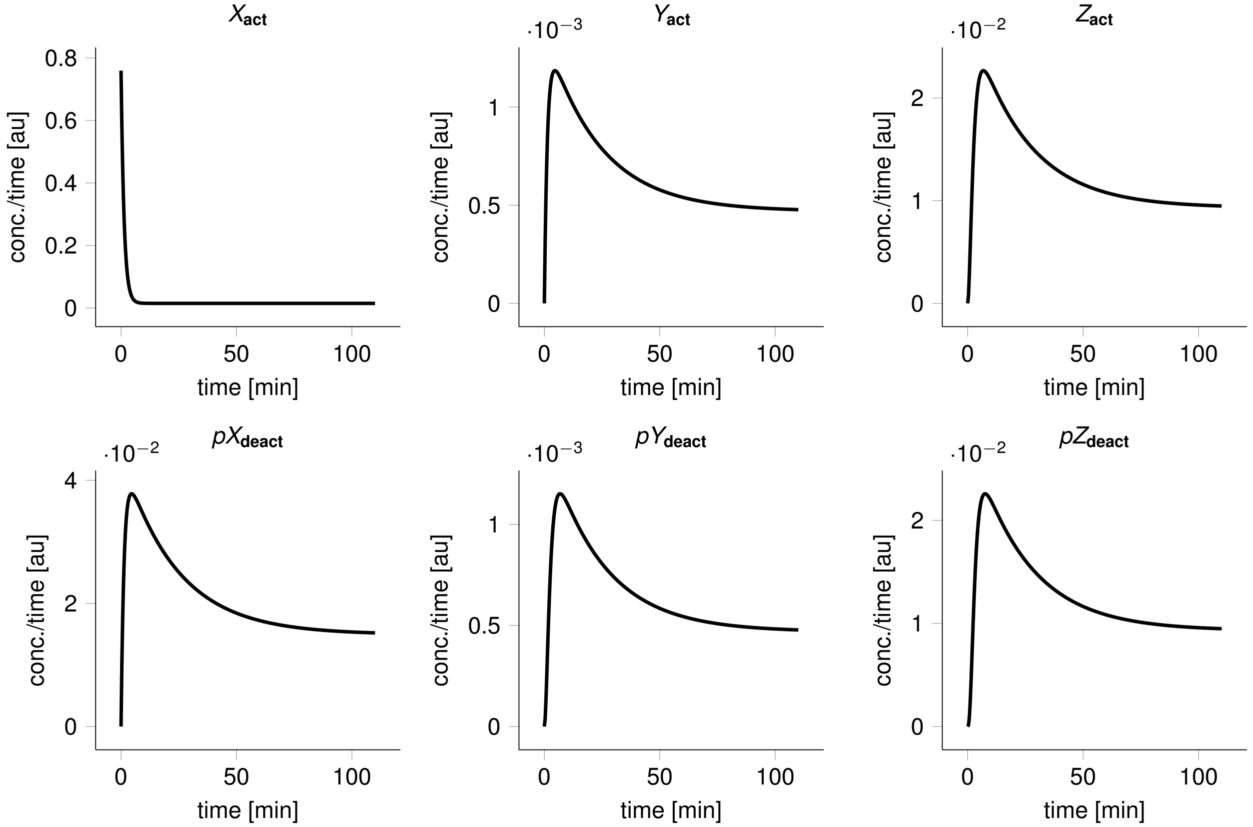

Figure 12: Flux analysis of scenario 3. The fluxes of both activation (upper) and deactivation (lower) of each particular state are on the same order of magnitude.

relation between Z and Y. This reduction leads to a significant worse fit of the data (Fig. 13), with a p-value of $p = 9.11 \times 10^{-}21$ resulting from the likelihood ratio test for one degree of freedom.

1.4 Scenario 4: $(- \updownarrow)$

1.4.1 Model scheme, equations and parameters

Reaction scheme:

$$X \underset{k\_off}{\overset{k\_on \cdot u(t)}{\rightleftharpoons}} pX$$

Equations:

$$\dot{X} = -k\_on \cdot X \cdot e^{-0.1 \cdot t} + k\_off \cdot pX$$
$$\dot{X} = k\_on \cdot X \cdot e^{-0.1 \cdot t} - k\_off \cdot pX$$

Observation:

$$pX\_obs = scale \cdot pX$$

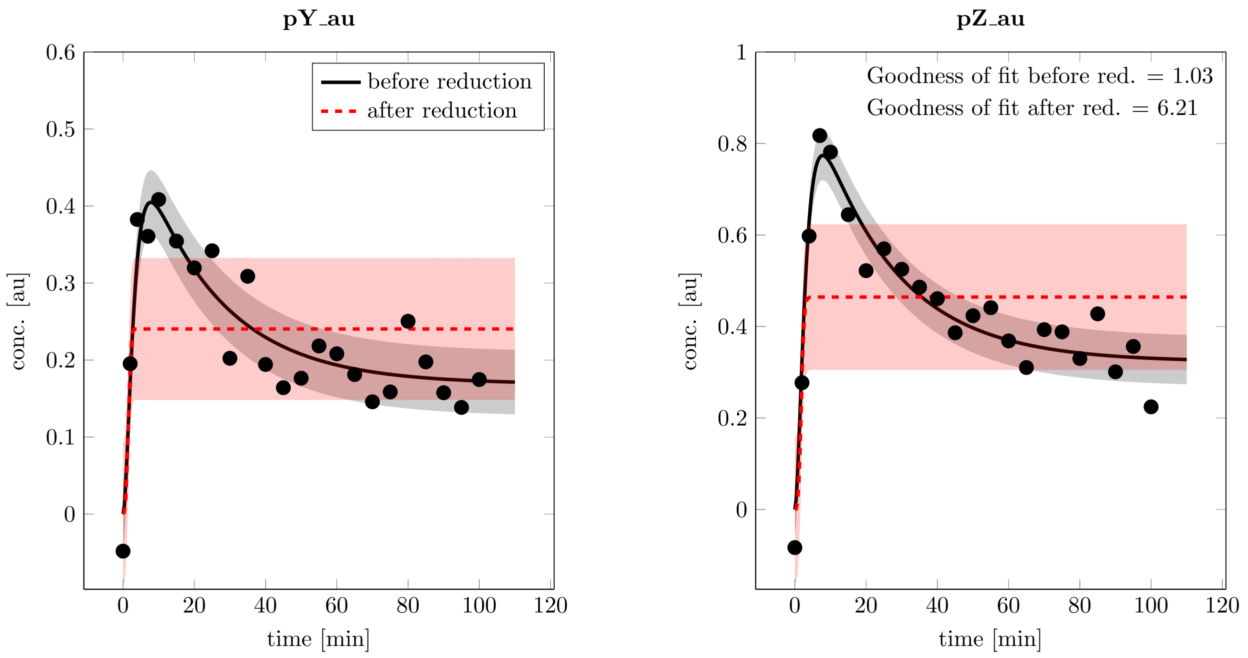

Figure 13: Reduction of `pY_deact` **in scenario 3.** The original fit of scenario 3 to the data (black trajectories) is compared to the model response after deletion of parameter `pY_deact` (red dotted trajectories), which is not proposed by the model reduction algorithm.

Parameters:

$$X(0) = 1$$
$$pX(0) = 0$$
$$k\_on = 0.01$$
$$k\_off = 0.1$$
$$scale = 10$$

1.4.2 Model definition

```
# Load libraries
library(deSolve)
library(parallel)
library(dMod)
```

```
# Generate reaction network
f <- NULL
f <- addReaction(f, "X", "pX", "k_on * X * exp(-0.1*time)")
f <- addReaction(f, "pX", "X", "k_off * pX")

# Define new observables based on ODE states
observables <- c(
  pX_obs = "scale * pX"
)
```

```r
# Generate observation function
g <- Y(observables, f, compile = TRUE, modelname = "obsfn2")

# Generate the model C files, compile them and return a list with func and extended.
model0 <- odemodel(f, compile = TRUE, modelname = "odefn2")

# Define inner parameters (parameters occurring in the equations except forcings)
innerpars <- union(getParameters(model0), getParameters(g))

# Define additional parameter constraints, e.g. initial states
constraints <- c(
  X = "1",
  pX = "0"
)

# Build up a parameter transformation (constraints, log-transform, etc.)
# Start with the identity
trafo <- structure(innerpars, names = innerpars)
# Then employ the other parameter constraints
trafo <- replaceSymbols(names(constraints), constraints, trafo)
# Then do a log-transform of all parameters (if defined as positive numbers)
trafo <- replaceSymbols(innerpars, paste0("exp(log", innerpars, ")"), trafo)
# Get names of new parameters
outerpars <- getSymbols(trafo)

# Generate parameter transformation function
p0 <- P(trafo, condition = "cond1")

# Generate prediction function
x0 <- Xs(model0)

# Generate prediction function with parameter transformation and observation function
y <- g*x0*p0
```

1.4.3 Simulation of Data

```r
# Use the following parameters
pouter <- c(logk_on = log(0.01),
            logk_off = log(0.1),
            logscale = log(10))

# Constant noise level and equidistant time points
noise <- 0.1
times <- seq(0, 40, by = 1.5)
set.seed(1)

# Predict model response and add noise
prediction <- y(times, pouter)
values.pA <- prediction$cond1[, "pX_obs"]
noise.pA <- rnorm(length(values.pA), 0, noise)
```

```
data <- datalist(
  cond1 = data.frame(name = "pX_obs",
                     time = times,
                     value = values.pA + noise.pA,
                     sigma = noise)
)
```

1.4.4 Model reduction analysis

```
# Initalize parameters
fixed <- c(logscale = 5)
pinit <- pouter[setdiff(outerpars, names(fixed))]

# Fit parameters and plot prediction and data
myfit <- trust(normL2(data, g*x0*p0), pinit, rinit = 1, rmax = 10, fixed = fixed)
plot(y(times, myfit$argument, fixed = fixed), data)
```

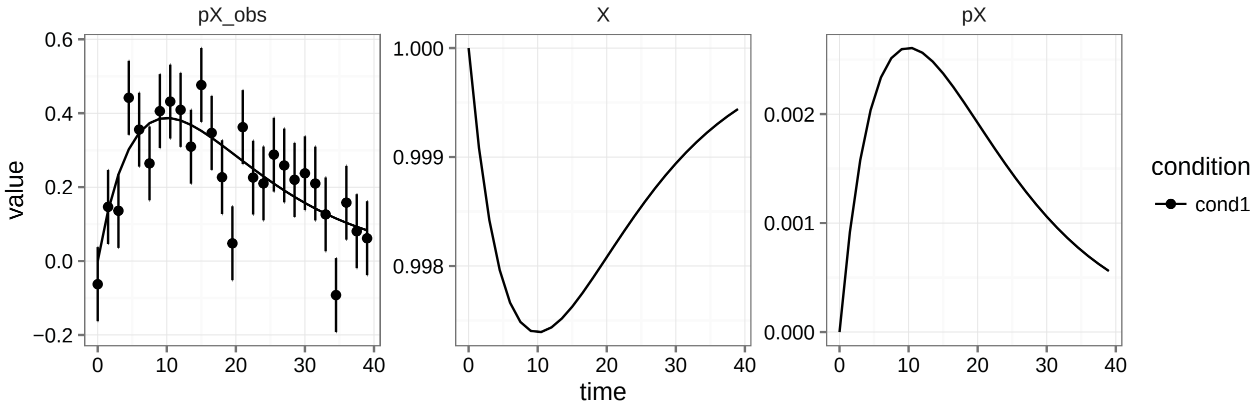

Figure 14: Model trajectories and data.

```
# Compute profile
bestfit <- c(myfit$argument, fixed)
myprof <- profile(normL2(data, g*x0*p0), pars = bestfit, whichPar = "logk_on", limit = c(-10, 10),
                  stepControl = list(max = .5, atol = 1e-1, rtol = 1e-1))

# Plot profile likelihood
plotProfile(myprof)
```

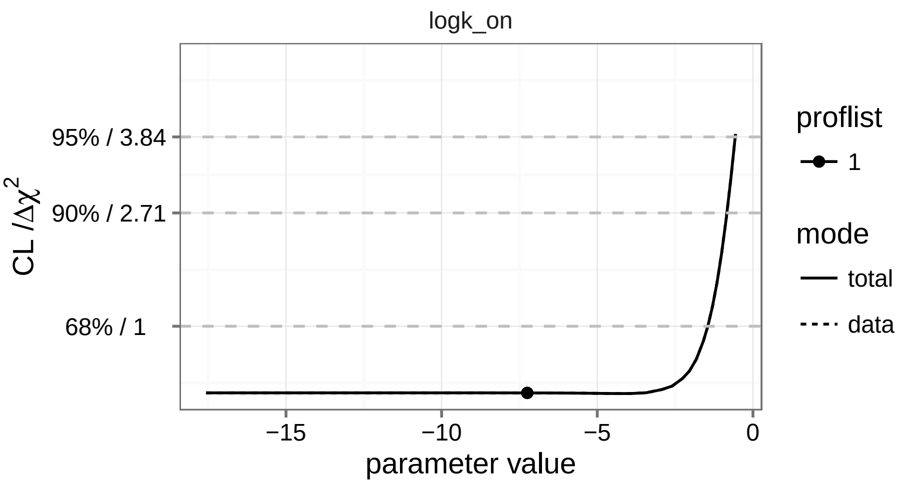

Figure 15: Profile likelihood of parameter k on.

```
# Plot associated parameter paths
plotPaths(myprof)
```

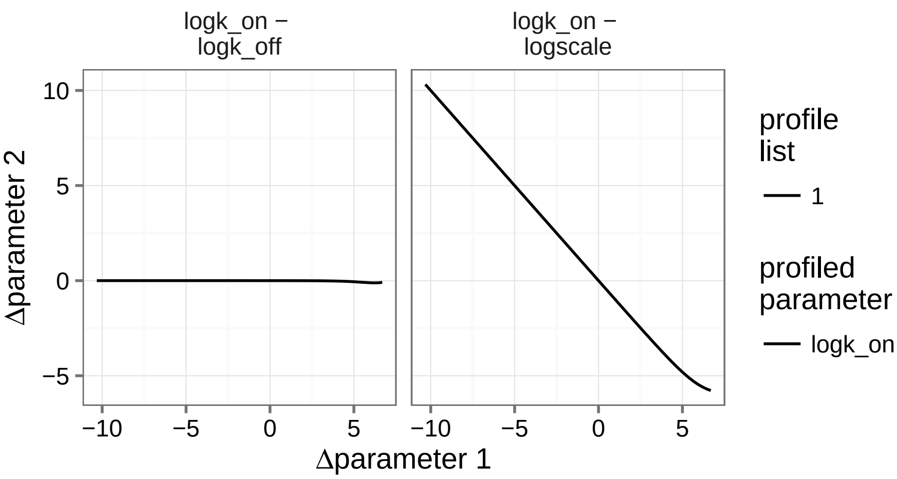

Figure 16: Associated parameter paths.

```
# Plot associated model predictions
plotArray(myprof, y, times, data)
```

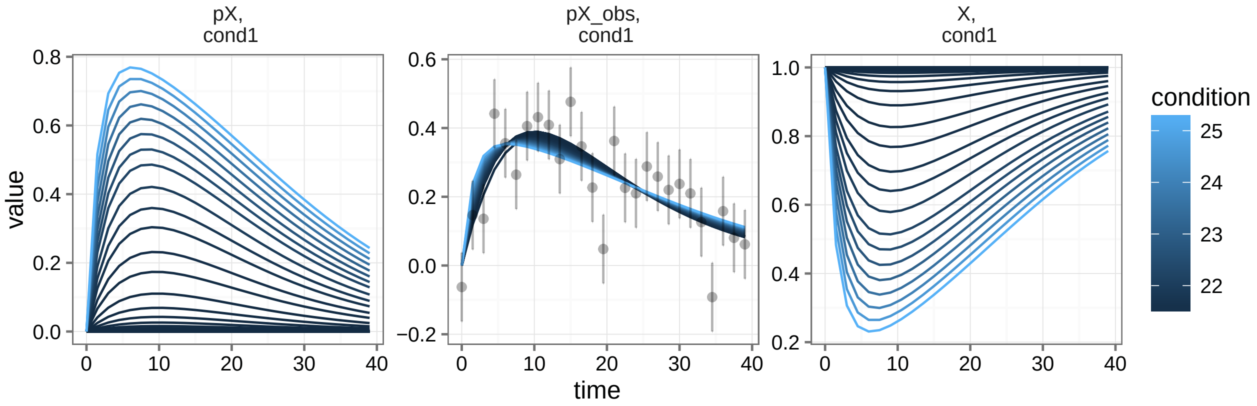

Figure 17: Associated model predictions.

1.4.5 Conclusion

In the limit $k_{on} \to 0$ the state A becomes a constant pool and the scaling parameter perfectly compensates the parameter k_{off}, i.e. scaling can be set to 1.

2 Model of reelin signalling pathway

2.1 Functioning of Reelin

The secreted protein Reelin has a dual role in the mammalian brain. It regulates the positioning and differentiation of postmitotic neurons during brain development and modulates neurotransmission and memory formation in the adult brain. Alterations in the Reelin signalling pathway have been described in different psychiatric disorders. Reelin mainly signals by binding to the lipoprotein receptors VLDLR and ApoER2, which induces tyrosine phosphorylation of the adaptor protein Dab1 by Src family kinases (SFKs). In turn, Dab1 activates the PI3-kinase-dependent signaling cascades [1, 2]. The model is depicted in Figure 18.

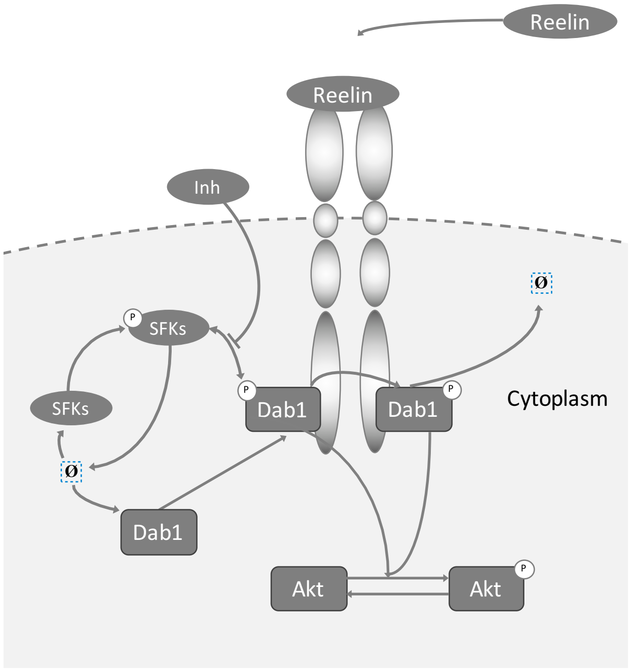

Figure 18: Scheme of the Reelin-induced signalling pathway. After binding of Reelin, clusters of the lipoprotein receptors ApoER2 and VLDLR are formed. These bind the adaptor protein Dab1, which is phosphorylated by SFKs. As a feed-forward loop, Dab1 can then trans-phosphorylate other Dab1 proteins bound to the receptor complex. In turn, phosphorylated Dab1 then activates Akt. The pathway is regulated by ubiquitination and degradation of phosphorylated Dab1.

2.2 Preparation and treatment of primary cortical neuron cultures

Cortical neurons were isolated from E15.5 NMRI wild-type mice, with E0.5 defined as the morning of plug detection and cultured as described in [3]. Cerebral cortices were dissected in Hanks' Balanced Salt Solution (HBSS) (Life Technologies) and trypsinized with 0.05 % trypsin-EDTA (Life Technologies) at 37 ° C for 25 min. Trypsinization was stopped by addition of an equal amount of FCS and cells were triturated and centrifuged at 500x g for 5 min. The cell pellet was resuspended in DMEM (4.5 g/l glucose) supplemented with 8 % FCS and 1x106 cells/ well were plated on 6-well plastic cell culture dishes coated with 0.05 mg/ml poly-D-lysine (Sigma). After 18 h the medium was replaced by serum-free Neurobasal medium supplemented with 2 % B27 and 1 mM GlutaMax (Life Technologies). Neurons were cultured for 5 days at 37 ° C and 5 % CO_2 and then stimulated with Reelin, at times pre-stimulated with the SFK tyrosine kinase inhibitor [4] PP2 (10 mM), or control-conditioned supernatant as described in [3] and analyzed by western blotting.

2.3 Western blotting

Cell lysates from neuron cultures were harvested in RIPA buffer (50 mM Tris-HCl pH 8.0, 1 % NP40, 150 mM NaCl, 0,1 % SDS, 1 mM EDTA pH 8,0, 12 mM sodium deoxycholate) supplemented with protease inhibitor (cOmplete ULTRA Tablets - Mini, EDTA-free, Roche) and phosphatase inhibitor (Phosphatase Inhibitor Cocktail 2 and 3, Sigma). Cell debris was removed from lysates by centrifugation (30 min at 17982x g, 4 ° C). Lysates were mixed with sample buffer (NuPAGE LDS Sample Buffer, Life Technologies) with 0,1 M DTT, heat-denatured, separated by SDS-PAGE and transferred to nitrocellulose membranes (GE Healthcare). For protein detection, membranes were incubated with the following primary antibodies: Mouse anti-ß-actin (1:5000, Abcam ab3280), rabbit anti-Dab1 (1:2000, Millipore MABS167), rabbit anti-p-Akt (1:1000, Cell Signaling 4060), rabbit anti-p-Src (1:1000, Cell Signaling 6943), mouse anti-p-tyrosine 4G10 (1:1000, Millipore 05-231) and respective secondary HRP-coupled antibodies (GE Healthcare, 1:7500). Detection was accomplished by using enhanced chemiluminescence and Fuji Super RX films. Densitometric quantification was done with ImageJ.

2.4 Mathematical modelling

A mechanistic model based on ordinary differential equations was built to model early dynamics in the Reelin-mediated signaling cascade. It was built within the freely available Data2Dynamics modelling environment described in [5] within MATLAB. The model was calibrated on time-resolved data of protein concentrations measured in cortical neurons treated with Reelin. In addition, an experimental condition with an SFK inhibitor, applied prior to Reelin stimulation, was included in the model. The measurements include the total Dab1 level as well as concentrations of phosphorylated Dab1, SFKs and Akt. For the initial concentrations of the model components, analytic steady state expressions were derived and are shown in Table 1 [6]. Based on this setup, the model reduction was performed according to the general recipes given in the main text. The 12 dynamic variables used in the model are summarised in Table 1. Their initial conditions were determined via steady state constraints, i.e. the time derivative of all dynamic variables have to be zero in the absence of a stimulus.

Variable	Unit	Initial Condition
ApoER2_Dab1	conc. [au]	Dab1_prod/(ApoER2_Dab1_SFK_bind*SFK_block)
ApoER2_Dab1SFK	conc. [au]	Dab1_prod/(ApoER2_Dab1_SFK_degrad*SFK_block)
ApoER2_Dab1SFKInh	conc. [au]	init_ApoER2_Dab1SFKInh
pApoER2_re_Dab1	conc. [au]	init_pApoER2_re_Dab1
ApoER2_re_Dab1	conc. [au]	init_ApoER2_re_Dab1
ApoER2_re_Dab1SFK	conc. [au]	init_ApoER2_re_Dab1SFK
ApoER2_re_Dab1SFKInh	conc. [au]	init_ApoER2_re_Dab1SFKInh
SFK_Int	conc. [au]	1
pSFK_Int	conc. [au]	0
Akt_Int	conc. [au]	init_Akt_Int
pAkt_Int	conc. [au]	(Akt_activation*Dab1_prod*init_Akt_Int)/ (Akt_deactivation*ApoER2_Dab1_SFK_degrad*SFK_block)

Table 1: Dynamic variables used in the model.

The time course of this 12 dynamic variables is gained by integration of the corresponding differential

equations, which are given by the following equations:

$$d[ApoER2\_Dab1]/dt = v_1 - v_4 - v_5 + v_7 - v_{11} - v_{12} - v_{14} \quad (1)$$

$$d[ApoER2\_Dab1SFK]/dt = v_4 - v_6 - v_8 \quad (2)$$

$$d[ApoER2\_Dab1SFKInh]/dt = v_5 - v_7 - v_9 \quad (3)$$

$$d[pApoER2\_re\_Dab1]/dt = v_{10} - v_{17} \quad (4)$$

$$d[ApoER2\_re\_Dab1]/dt = -v_{10} + v_{11} - v_{13} - v_{15} + v_{16} \quad (5)$$

$$d[ApoER2\_re\_Dab1SFK]/dt = v_8 + v_{12} + v_{13} - v_{18} \quad (6)$$

$$d[ApoER2\_re\_Dab1SFKInh]/dt = v_9 + v_{14} + v_{15} - v_{16} \quad (7)$$

$$d[SFK\_Int]/dt = -v_2 + v_3 - v_4 + v_6 - v_{12} - v_{13} \quad (8)$$

$$d[SFK\_Inh]/dt = v_2 - v_3 - v_5 + v_7 - v_{14} - v_{15} + v_{16} \quad (9)$$

$$d[Akt\_Int]/dt = -v_{19} + v_{20} \quad (10)$$

$$d[pAkt\_Int]/dt = v_{19} - v_{20} \quad (11)$$

The flux expressions corresponding to these equations are provided in Table 2. The system of ODEs was integrated using a multi-threaded implementation of the CVODES algorithm [7, 8]. First order derivatives were computed using the sensitivity equations and used for numerical optimization [9]. Relative and absolute tolerances were set to 1e-06.

Flux	Equation
v_1	Dab1_prod
v_2	Inh · SFK_Inhib · [SFK_Int]
v_3	[SFK_Inh] · SFK_reInhib
v_4	[ApoER2_Dab1] · ApoER2_Dab1_SFK_bind · [SFK_Int] · SFK_block
v_5	[ApoER2_Dab1] · ApoER2_Dab1_SFK_bind · [SFK_Inh] · SFK_block
v_6	[ApoER2_Dab1SFK] · ApoER2_Dab1_SFK_degrad · SFK_block
v_7	[ApoER2_Dab1SFKInh] · ApoER2_Dab1_SFK_unbind · SFK_block
v_8	[ApoER2_Dab1SFK] · ApoER2_bindReelin · [Reelin]
v_9	[ApoER2_Dab1SFKInh] · ApoER2_bindReelin · [Reelin]
v_{10}	ApoER2_Dab1_act · [ApoER2_re_Dab1] · [ApoER2_re_Dab1SFK]
v_{11}	[ApoER2_Dab1] · ApoER2_bindReelin · [Reelin]
v_{12}	[ApoER2_Dab1] · ApoER2_Dab1_SFK_bind · [Reelin] · [SFK_Int]
v_{13}	ApoER2_Dab1_SFK_bind · [ApoER2_re_Dab1] · [SFK_Int]
v_{14}	[ApoER2_Dab1] · ApoER2_Dab1_SFK_bind · [Reelin] · [SFK_Inh]
v_{15}	ApoER2_Dab1_SFK_bind · [ApoER2_re_Dab1] · [SFK_Inh]
v_{16}	ApoER2_Dab1_SFK_unbind · [ApoER2_re_Dab1SFKInh]
v_{17}	[pApoER2_re_Dab1] · pApoER2_re_Dab1_deact
v_{18}	[ApoER2_re_Dab1SFK] · ApoER2_re_Dab1SFK_degrad
v_{19}	[Akt_Int] · Akt_activation · ([ApoER2_Dab1SFK] + [ApoER2_re_Dab1SFK] + [pApoER2_re_Dab1])
v_{20}	Akt_deactivation · [pAkt_Int]

Table 2: Model flux expressions

2.5 Dynamic parameters

In total 29 parameters are estimated from the experimental data, yielding a value of the objective function $\chi^2 = 93.9237$ for a total of 108 data points. The model parameters were estimated by maximum likelihood estimation applying the MATLAB lsqnonlin algorithm [10] and a deterministic multi-start optimisation strategy [11]. The model parameters which influence system dynamics are listed in Table 3.

	name	θ_{min}	$\hat{\theta}$	θ_{max}	log	non-log $\hat{\theta}$	fitted
1	Akt_activation	-5	-2.7771	+0	1	$+1.67 \cdot 10^{-03}$	1
2	Akt_deactivation	-5	+0.0480	+3	1	$+1.12 \cdot 10^{+00}$	1
3	ApoER2_Dab1_SFK_bind	-5	-3.4989	+3	1	$+3.17 \cdot 10^{-04}$	1
4	ApoER2_Dab1_SFK_degrad	-5	+1.9577	+3	1	$+9.07 \cdot 10^{+01}$	1
5	ApoER2_Dab1_SFK_unbind	-5	-0.9680	+3	1	$+1.08 \cdot 10^{-01}$	1
6	ApoER2_Dab1_act	-5	-1.1937	+3	1	$+6.40 \cdot 10^{-02}$	1
7	ApoER2_bindReelin	-5	-1.7736	+3	1	$+1.68 \cdot 10^{-02}$	1
8	ApoER2_re_Dab1SFK_degrad	-5	-2.0224	+3	1	$+9.50 \cdot 10^{-03}$	1
9	Dab1_prod	-2	+1.0861	+3	1	$+1.22 \cdot 10^{+01}$	1
10	SFK_Inhib	-5	-0.5317	+2	1	$+2.94 \cdot 10^{-01}$	1
11	SFK_block	-3	+1.0000	+3	0	$+1.00 \cdot 10^{+00}$	2
12	SFK_reInhib	-5	-2.1091	+3	1	$+7.78 \cdot 10^{-03}$	1
13	init_Akt_Int	-5	-1.0000	+3	1	$+1.00 \cdot 10^{-01}$	2
14	init_ApoER2_Dab1SFKInh	-2	+0.0000	+2	0	$+0.00 \cdot 10^{+00}$	2
15	init_ApoER2_re_Dab1	-2	+0.0000	+2	0	$+0.00 \cdot 10^{+00}$	2
16	init_ApoER2_re_Dab1SFK	-2	+0.0000	+2	0	$+0.00 \cdot 10^{+00}$	2
17	init_ApoER2_re_Dab1SFKInh	-2	+0.0000	+2	0	$+0.00 \cdot 10^{+00}$	2
18	init_SFK_Inh	-2	+0.0000	+2	0	$+0.00 \cdot 10^{+00}$	2
19	init_pApoER2_re_Dab1	-2	+0.0000	+2	0	$+0.00 \cdot 10^{+00}$	2
28	pApoER2_re_Dab1_deact	-5	+2.8927	+3	1	$+7.81 \cdot 10^{+02}$	1

Table 3: Estimated dynamic parameter values
$\hat{\theta}$ indicates the estimated value of the parameters. θ_{min} and θ_{max} indicate the upper and lower bounds for the parameters. The log-column indicates if the value of a parameter was log-transformed. If log = 1 the non-log-column indicates the non-logarithmic value of the estimate. The fitted-column indicates if the parameter value was estimated (1), was temporarily fixed (0) or if its value was fixed to a constant value (2).

2.6 Model fit and plots

The model observables and the experimental data is shown in Figure 19. The agreement of the model

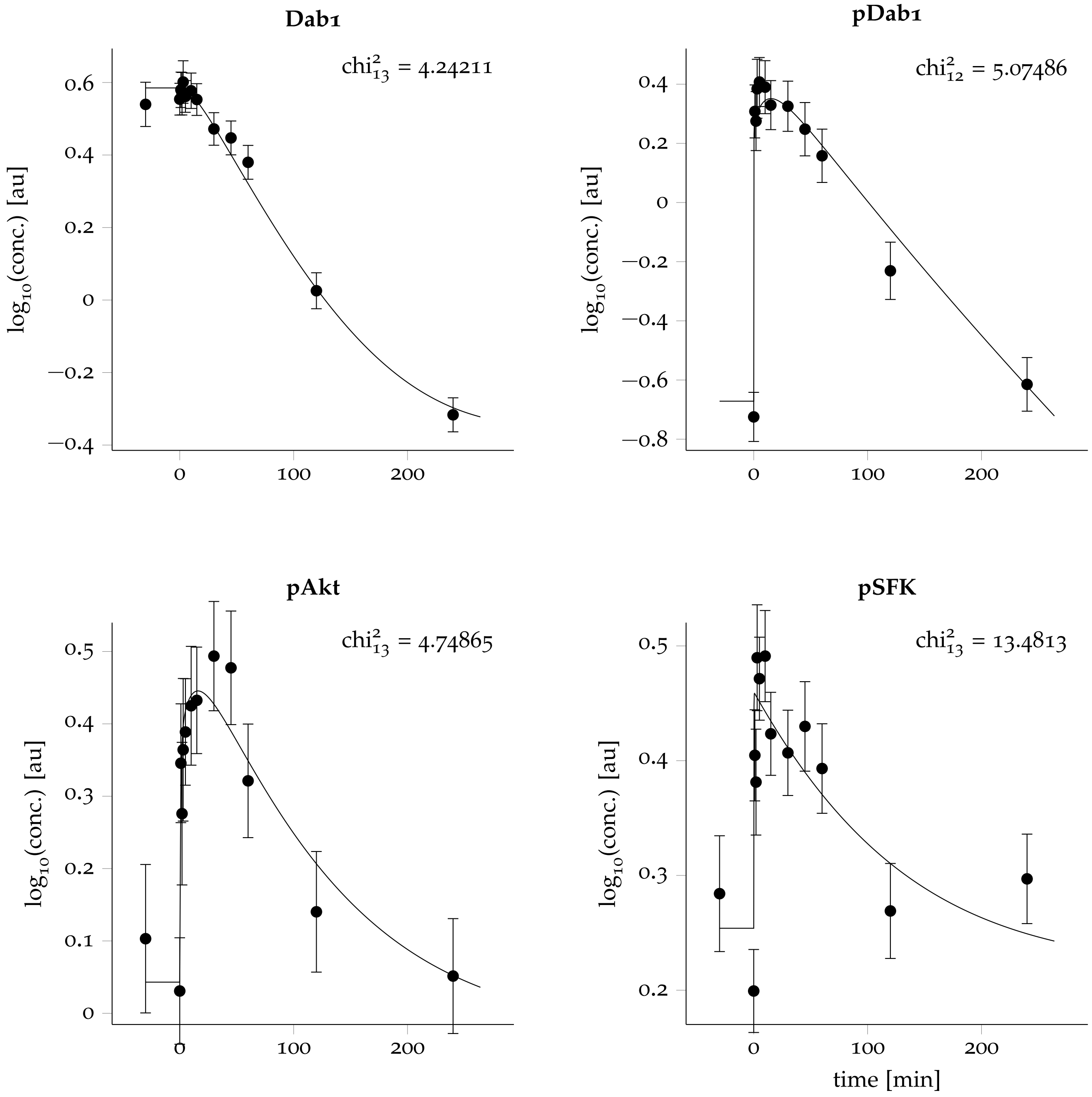

Figure 19: Observables and experimental data for the experiment with Reelin stimulation. The observables are displayed as solid lines. The data points with corresponding error are shown as black dots with error bars.

observables and the experimental data, given in Table 4 and 5, yields a value of the objective function $\chi^2 = 47.3332$ for 73 data points in this data set.

time [min]	Reelin_input	Inh	Dab1 conc. [au]	Dab1_std conc. [au]	pDab1 conc. [au]	pDab1_std conc. [au]
-30	1	0	3.46461	0.0610049	NaN	NaN
0	1	0	3.58099	0.0437671	0.188505	0.0830433
1	1	0	3.80049	0.04883	2.03231	0.0895569
2	1	0	3.70742	0.0586007	1.8847	0.0996509
3	1	0	3.99311	0.0586007	2.42401	0.0996509
5	1	0	3.64345	0.0437671	2.55493	0.082842
10	1	0	3.77753	0.04883	2.45468	0.0895569
15	1	0	3.57258	0.0437671	2.13457	0.082842
30	1	0	2.96395	0.0449246	2.11596	0.0848068
45	1	0	2.7999	0.0466793	1.7698	0.0900869
60	1	0	2.3978	0.0466793	1.4387	0.0900869
120	1	0	1.06076	0.0495766	0.587694	0.0968194
240	1	0	0.482395	0.0469452	0.242811	0.0906662

Table 4: Experimental data for the experiment with Reelin stimulation

time [min]	Reelin_input	Inh	pSFK conc. [au]	pSFK_std conc. [au]	pAkt conc. [au]	pAkt_std conc. [au]
-30	1	0	1.92367	0.0504568	1.26791	0.102561
0	1	0	1.58236	0.036162	1.07353	0.0735771
1	1	0	2.53908	0.0397192	2.216	0.0820903
2	1	0	2.40615	0.0461281	1.88758	0.0985136
3	1	0	3.08814	0.0461281	2.31295	0.0985136
5	1	0	2.96109	0.036162	2.44782	0.0735771
10	1	0	3.09835	0.0397192	2.66059	0.0820903
15	1	0	2.65094	0.036162	2.70696	0.0735771
30	1	0	2.55171	0.0371645	3.11609	0.0755248
45	1	0	2.69071	0.0389916	3.00214	0.0784729
60	1	0	2.47263	0.0389916	2.09528	0.0784729
120	1	0	1.85804	0.0413763	1.3812	0.0833467
240	1	0	1.98162	0.0389916	1.12577	0.079397

Table 5: Experimental data for the experiment with Reelin stimulation

2.7 Experimental condition with SFK inhibition

The model observables and the experimental data is shown in Figure 20. The agreement of the model

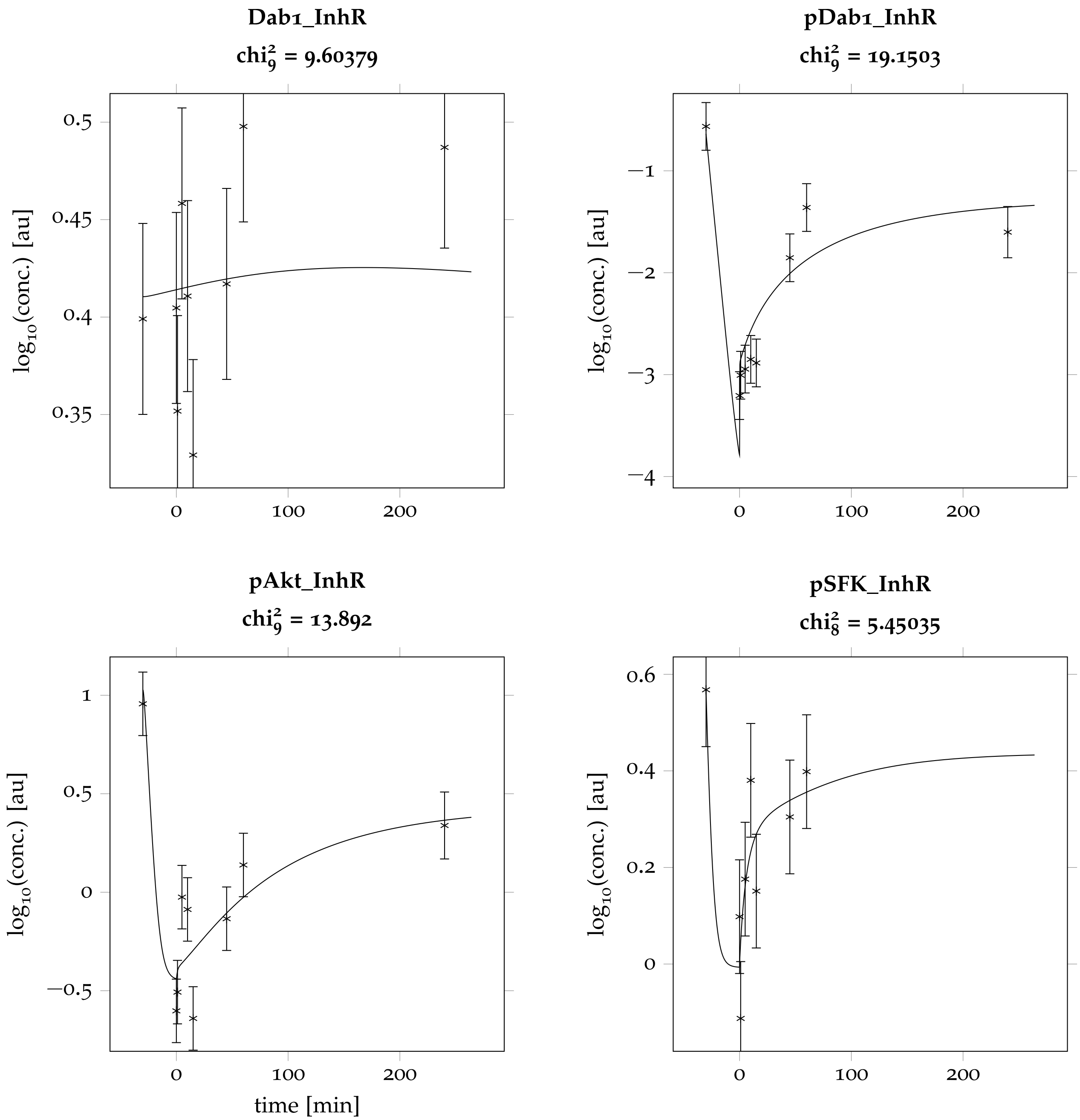

Figure 20: Reelin with SFK inhibitor observables and experimental data. The observables are displayed as solid lines. The data points with corresponding error are shown as black dots with error bars.

observables and the experimental data, given in Table 7 and 7, yields a value of the objective function $\chi^2 = 48.0964$ for 35 data points in this data set.

time [min]	Reelin_input	Inh	Dab1_InhR conc. [au]	Dab1_InhR_std conc. [au]	pDab1_InhR conc. [au]	pDab1_InhR_std conc. [au]
-30	1	1	2.50644	0.0489828	0.272812	0.234157
0	1	1	2.53914	0.0489828	0.000622577	0.234157
1	1	1	2.24789	0.0489828	0.000986718	0.234157
5	1	1	2.87282	0.0489828	0.00113344	0.234157
10	1	1	2.57495	0.0489828	0.00141196	0.234157
15	1	1	2.13412	0.0489828	0.00130198	0.234157
45	1	1	2.61222	0.0489828	0.0140223	0.234157
60	1	1	3.14637	0.0489828	0.0436419	0.234157
240	1	1	3.06944	0.05166	0.0250261	0.251223

Table 6: Experimental data for the experiment Reelin with SFK inhibitor.

time [min]	Reelin_input	Inh	pSFK_InhR conc. [au]	pSFK_InhR_std conc. [au]	pAkt_InhR conc. [au]	pAkt_InhR_std conc. [au]
-30	1	1	3.69723	0.11771	9.04763	0.16111
0	1	1	1.25339	0.11771	0.249891	0.16111
1	1	1	0.771516	0.11771	0.311134	0.16111
5	1	1	1.49891	0.11771	0.944299	0.16111
10	1	1	2.401	0.11771	0.817949	0.16111
15	1	1	1.41569	0.11771	0.22874	0.16111
45	1	1	2.01633	0.11771	0.733818	0.16111
60	1	1	2.50263	0.11771	1.37511	0.16111
240	1	1	NaN	NaN	2.18246	0.169916

Table 7: Experimental data for the experiment Reelin with SFK inhibitor.

2.8 Parameter profiles

For the given model, parameter profiles are obtained [12, 13]. The profile of the Akt deactivation does not exceed the 95 % threshold for large values, with adapting Akt activation (Figure 21). As described in the main text, both parameters going to infinity without feedbacks within the model will result in an Akt time course in parallel to the time course of the upstream protein, in this case pDab1 [14]. Thus, the model reduction leads to $pAkt = scale_{pAkt}\, pDab1$, with identifiable scaling factor (Figure 22). Computation of the profiles took 34 s on average on a MacBookPro from Mid-2014 with a 2.8-GhZ Intel Core i5.

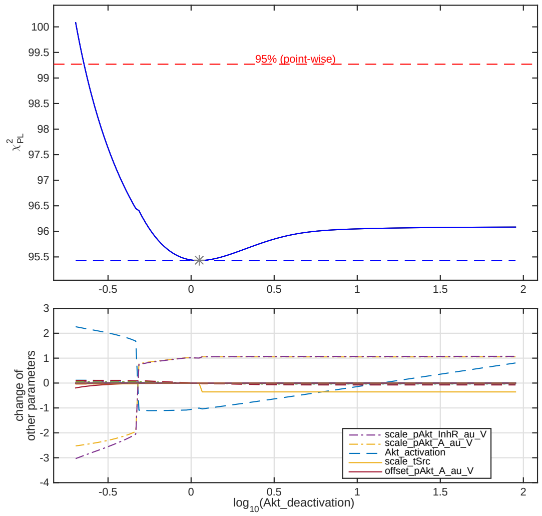

Figure 21: PLE of Akt deactivation. Profile likelihood of dynamic variable for Akt deactivation, which does not exceed the 95 % threshold for large parameter values. In addition, a linear relation to the Akt activation can be observed.

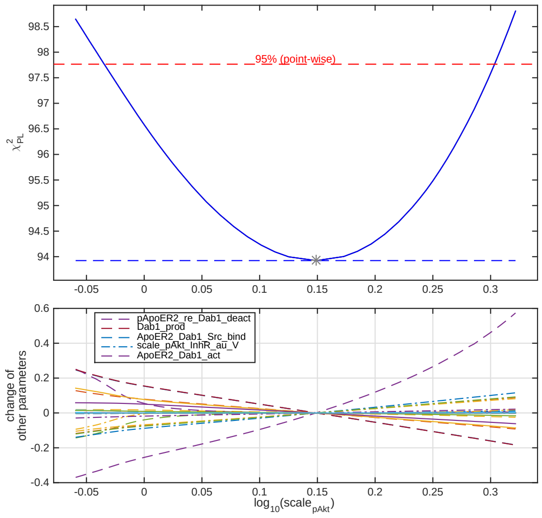

Figure 22: PLE of identifiable scaling factor determining the ratio between pAkt and pDab1.

In addition, an experiment with a SFK inhibitor prior to Reelin stimulation was exerted. Thereby, the profiles of the parameters for binding and release of the inhibitor to the SFKs do not exceed the 95 % threshold for parameter values going to infinity (Figure 23). With both parameters going to high values in parallel, the system describes an infinitely fast achieved steady state with fix partitioning between both states, with and without inhibitor. However, their ratio is identifiable (Figure 24).

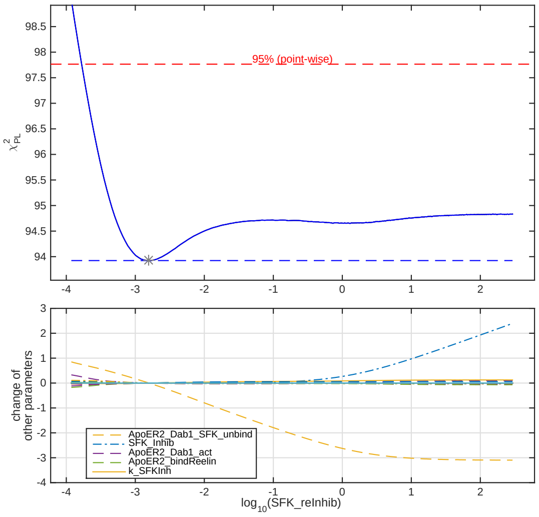

Figure 23: PLE of the release of the SFK inhibitor. The release of the SFK inhibitor can take large values without leading to a χ^2 above the 95 % threshold, with linear relation to the docking of the inhibitor.

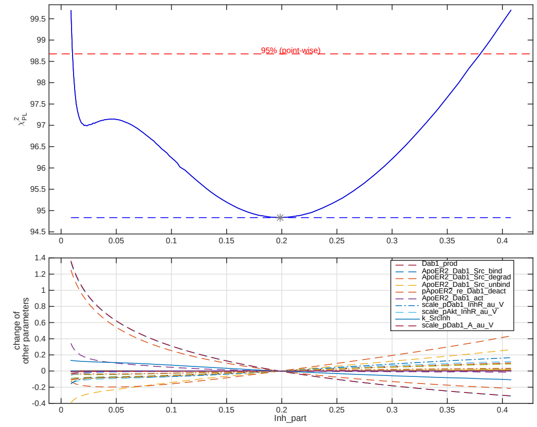

Figure 24: PLE of identifiable partition scale between SFKs with and without bound inhibitor.

3 Model after full reduction

After performing the model reduction, the ODEs have changed to the following:

$$d[ApoER2\_Dab1]/dt = \nu_1 - \nu_2 - \nu_3 + \nu_5 - \nu_9 - \nu_{10} - \nu_{12} \tag{12}$$
$$d[ApoER2\_Dab1Src]/dt = \nu_2 - \nu_4 - \nu_6 \tag{13}$$
$$d[ApoER2\_Dab1SrcInh]/dt = \nu_3 - \nu_5 - \nu_7 \tag{14}$$
$$d[pApoER2\_re\_Dab1]/dt = \nu_8 - \nu_{15} \tag{15}$$
$$d[ApoER2\_re\_Dab1]/dt = -\nu_8 + \nu_9 - \nu_{11} - \nu_{13} + \nu_{14} \tag{16}$$
$$d[ApoER2\_re\_Dab1Src]/dt = \nu_6 + \nu_{10} + \nu_{11} - \nu_{16} \tag{17}$$
$$d[ApoER2\_re\_Dab1SrcInh]/dt = \nu_7 + \nu_{12} + \nu_{13} - \nu_{14} \tag{18}$$
$$d[Src\_Int]/dt = -\nu_2 - \nu_3 + \nu_4 + \nu_5 - \nu_{10} - \nu_{11} - \nu_{12} - \nu_{13} + \nu_{14} \tag{19}$$
$$d[pSrc\_Int]/dt = \tag{20}$$

with flux expressions given in Table 8. Due to the reduction of pAkt as algebraic expression of pDab1, there is no explicit Akt and pAkt state left in the integration.

Flux	Equation
ν_1	Dab1_prod
ν_2	−[ApoER2_Dab1] · ApoER2_Dab1_SFK_bind · [SFK_Int] · SFK_block · (Inh · alpha − 1)
ν_3	[ApoER2_Dab1] · ApoER2_Dab1_SFK_bind · Inh · [SFK_Int] · SFK_block · alpha
ν_4	[ApoER2_Dab1SFK] · ApoER2_Dab1_SFK_degrad · SFK_block
ν_5	[ApoER2_Dab1SFKInh] · ApoER2_Dab1_SFK_unbind · SFK_block
ν_6	[ApoER2_Dab1SFK] · ApoER2_bindReelin · [Reelin]
ν_7	[ApoER2_Dab1SFKInh] · ApoER2_bindReelin · [Reelin]
ν_8	ApoER2_Dab1_act · [ApoER2_re_Dab1] · [ApoER2_re_Dab1SFK]
ν_9	[ApoER2_Dab1] · ApoER2_bindReelin · [Reelin]
ν_{10}	−[ApoER2_Dab1] · ApoER2_Dab1_SFK_bind · [Reelin] · [SFK_Int] · (Inh · alpha − 1)
ν_{11}	−ApoER2_Dab1_SFK_bind · [ApoER2_re_Dab1] · [SFK_Int] · (Inh · alpha − 1)
ν_{12}	[ApoER2_Dab1] · ApoER2_Dab1_SFK_bind · Inh · [Reelin] · [SFK_Int] · alpha
ν_{13}	ApoER2_Dab1_SFK_bind · [ApoER2_re_Dab1] · Inh · [SFK_Int] · alpha
ν_{14}	ApoER2_Dab1_SFK_unbind · [ApoER2_re_Dab1SFKInh]
ν_{15}	[pApoER2_re_Dab1] · pApoER2_re_Dab1_deact
ν_{16}	[ApoER2_re_Dab1SFK] · ApoER2_re_Dab1SFK_degrad

Table 8: Model flux expressions

3.1 Dynamic parameters

In total 24 parameters are estimated from the experimental data, yielding a value of the objective function $\chi^2 = 94.8366$ for a total of 108 data points. Hence, the amount of parameters is reduced by five, leading to a change in χ^2 of roughly 1. The new model parameters which influence system dynamics are listed in Table 9.

	name	θ_{min}	$\hat{\theta}$	θ_{max}	log	non-log $\hat{\theta}$	fitted
1	ApoER2_Dab1_SFK_bind	-7	-4.4499	+3	1	$+3.55 \cdot 10^{-05}$	1
2	ApoER2_Dab1_SFK_degrad	-5	+1.0621	+3	1	$+1.15 \cdot 10^{+01}$	1
3	ApoER2_Dab1_SFK_unbind	-7	-4.1257	+3	1	$+7.49 \cdot 10^{-05}$	1
4	ApoER2_Dab1_act	-5	-1.2759	+3	1	$+5.30 \cdot 10^{-02}$	1
5	ApoER2_bindReelin	-5	-1.7703	+3	1	$+1.70 \cdot 10^{-02}$	1
6	ApoER2_re_Dab1SFK_degrad	-5	-1.9990	+3	1	$+1.00 \cdot 10^{-02}$	1
7	Dab1_prod	-2	+0.1306	+3	1	$+1.35 \cdot 10^{+00}$	1
8	SFK_block	-3	+1.0000	+3	0	$+1.00 \cdot 10^{+00}$	2
9	alpha	+0	+0.1982	+1	0	$+1.98 \cdot 10^{-01}$	1
10	init_ApoER2_Dab1SFKInh	-2	+0.0000	+2	0	$+0.00 \cdot 10^{+00}$	2
11	init_ApoER2_re_Dab1	-2	+0.0000	+2	0	$+0.00 \cdot 10^{+00}$	2
12	init_ApoER2_re_Dab1SFK	-2	+0.0000	+2	0	$+0.00 \cdot 10^{+00}$	2
13	init_ApoER2_re_Dab1SFKInh	-2	+0.0000	+2	0	$+0.00 \cdot 10^{+00}$	2
14	init_pApoER2_re_Dab1	-2	+0.0000	+2	0	$+0.00 \cdot 10^{+00}$	2
23	pApoER2_re_Dab1_deact	-5	+2.9769	+6	1	$+9.48 \cdot 10^{+02}$	1

Table 9: Estimated dynamic parameter values
$\hat{\theta}$ indicates the estimated value of the parameters. θ_{min} and θ_{max} indicate the upper and lower bounds for the parameters. The log-column indicates if the value of a parameter was log-transformed. If log = 1 the non-log-column indicates the non-logarithmic value of the estimate. The fitted-column indicates if the parameter value was estimated (1), was temporarily fixed (0) or if its value was fixed to a constant value (2).

After reduction, the time courses have changed slightly as shown in Figures 25 and 26.

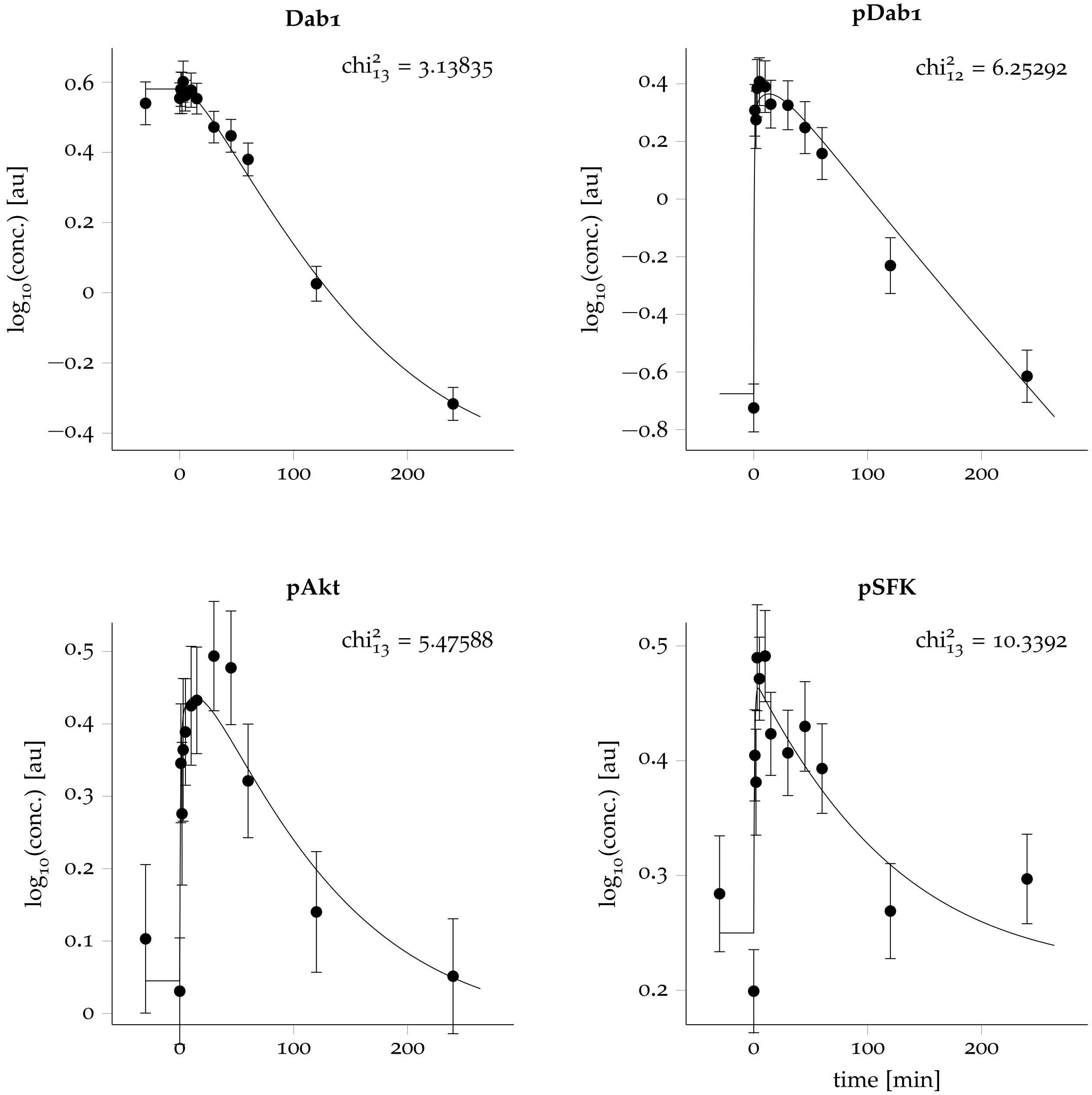

Figure 25: Observables and experimental data for the experiment with Reelin stimulation after model reduction. The observables are displayed as solid lines. The data points with corresponding error are shown as black dots with error bars.

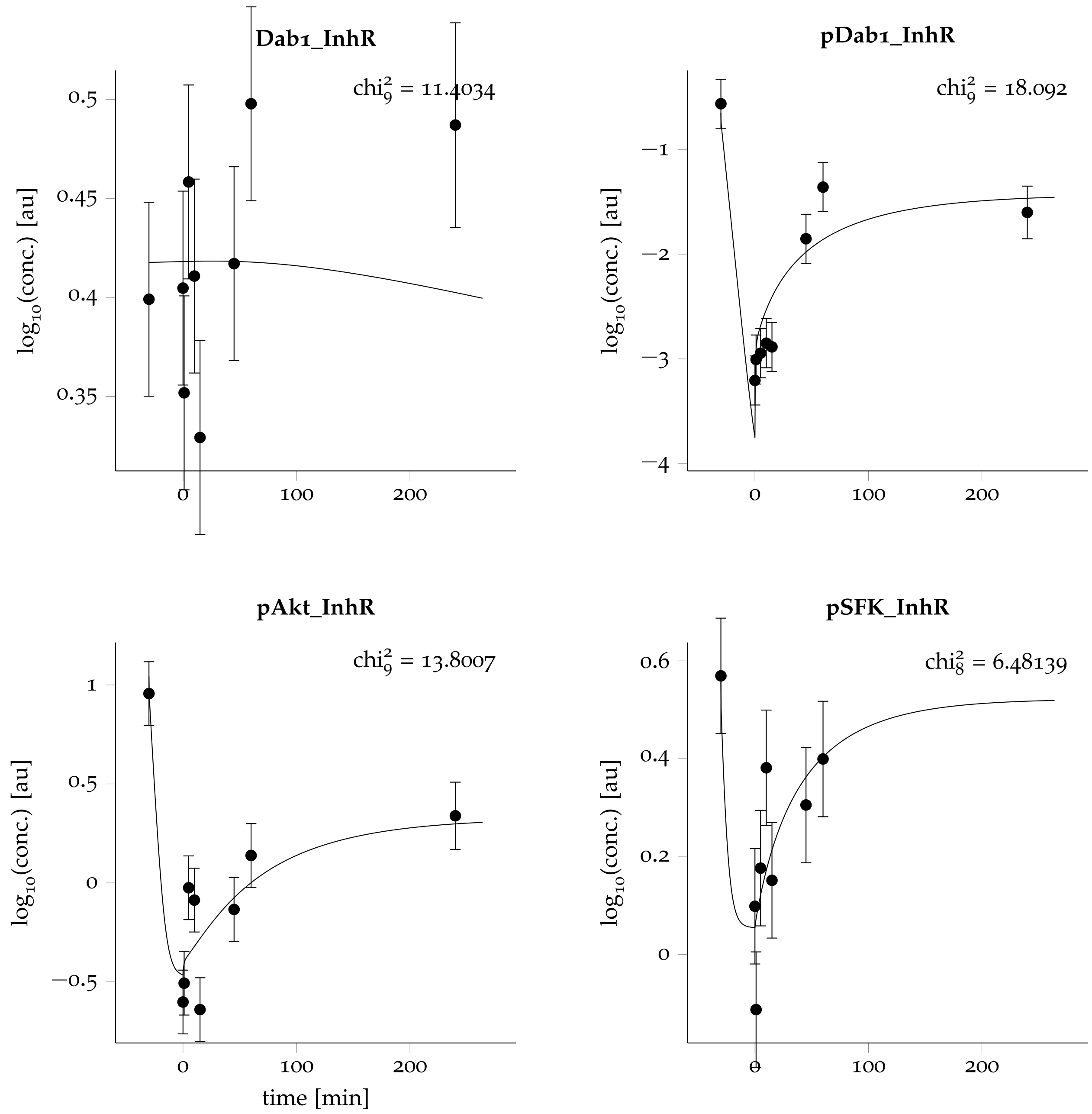

Figure 26: Reelin with SFK inhibitor observables and experimental data after model reduction. The observables are displayed as solid lines. The data points with corresponding error are shown as black dots with error bars.

Further, all parameter profiles are now identifiable, which can be seen by all profiles exceeding the 95 % threshold in both directions (Figure 27).

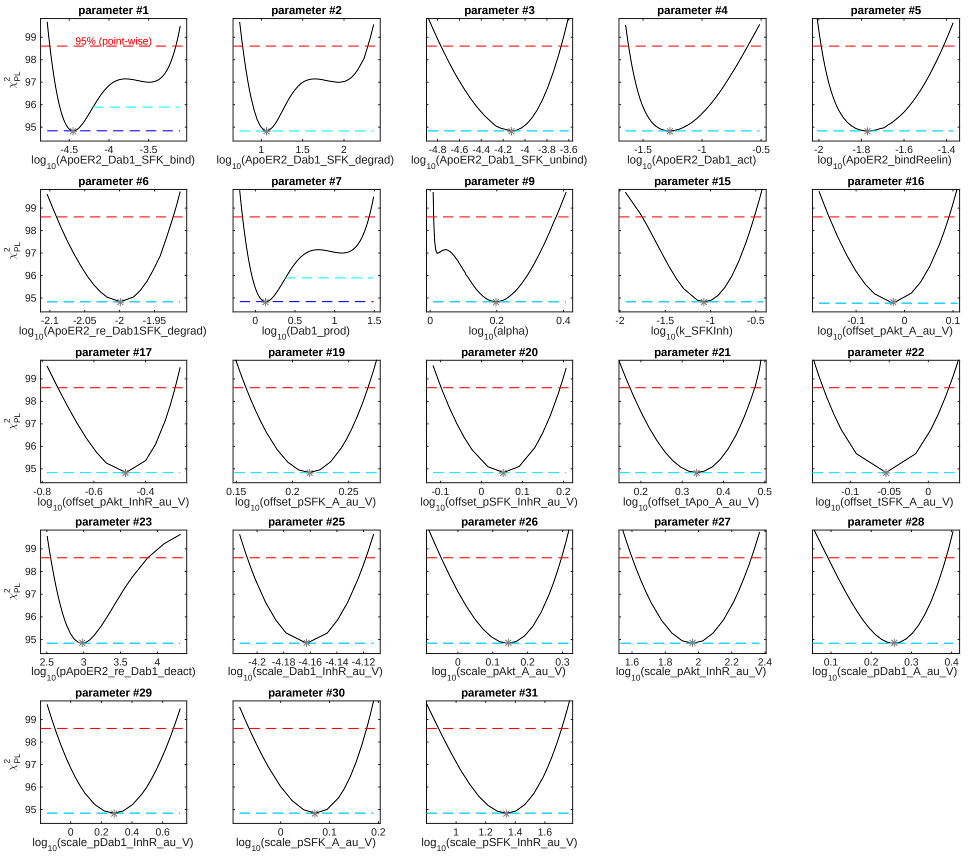

Figure 27: PLEs of all model parameters. The reduced model is fully identifiable.

References

1. Bock HH, Herz J. Reelin activates SRC family tyrosine kinases in neurons. Current Biology. 2003;13(1):18–26.

2. Feng L, Cooper JA. Dual functions of Dab1 during brain development. Molecular and Cellular Biology. 2009;29(2):324–332.

3. Bouché E, Romero-Ortega MI, Henkemeyer M, Catchpole T, Leemhuis J, Frotscher M, et al. Reelin induces EphB activation. Cell research. 2013;23(4):473–490.

4. Hanke JH, Gardner JP, Dow RL, Changelian PS, Brissette WH, Weringer EJ, et al. Discovery of a novel, potent, and Src family-selective tyrosine kinase inhibitor Study of Lck-and FynT-dependent T cell activation. Journal of Biological Chemistry. 1996;271(2):695–701.

5. Raue A, Steiert B, Schelker M, Kreutz C, Maiwald T, Hass H, et al. Data2Dynamics: a modeling environment tailored to parameter estimation in dynamical systems. Bioinformatics. 2015;31(21):3558–3560.

6. Segel IH, Segel AH. Biochemical Calculations: How to solve mathematical Problems in General Biochemistry. Wiley; 1968.

7. Hindmarsh AC, Brown PN, Grant KE, Lee SL, Serban R, Shumaker DE, et al. SUNDIALS: Suite of nonlinear and differential/algebraic equation solvers. ACM Transactions on Mathematical Software (TOMS). 2005;31(3):363–396.

8. Serban R, Hindmarsh AC. CVODES: the sensitivity-enabled ODE solver in SUNDIALS. In: ASME 2005 International Design Engineering Technical Conferences and Computers and Information in Engineering Conference. American Society of Mechanical Engineers; 2005. p. 257–269.

9. Leis JR, Kramer MA. The simultaneous solution and sensitivity analysis of systems described by ordinary differential equations. ACM Transactions on Mathematical Software (TOMS). 1988;14(1):45–60.

10. Coleman TF, Li Y. An interior, trust region approach for nonlinear minimization subject to bounds. SIAM Journal on Optimization. 1996;6:418–445.

11. Raue A, Schilling M, Bachmann J, Matteson A, Schelker M, Kaschek D, et al. Lessons learned from quantitative dynamical modeling in systems biology. PloS ONE. 2013;8(9):e74335.

12. Raue A, Kreutz C, Maiwald T, Bachmann J, Schilling M, Klingmüller U, et al. Structural and practical identifiability analysis of partially observed dynamical models by exploiting the profile likelihood. Bioinformatics. 2009;25(15):1923–1929.

13. Venzon D, Moolgavkar S. A method for computing profile-likelihood-based confidence intervals. Applied Statistics. 1988;37(1):87–94.

14. Heinrich R, Neel BG, Rapoport TA. Mathematical models of protein kinase signal transduction. Molecular Cell. 2002;9(5):957–970.
